# Organotypic artery-graft culture enables label-free multiphoton tracking of remodeling that links to long-term graft microarchitecture

**DOI:** 10.64898/2026.02.28.708759

**Published:** 2026-03-03

**Authors:** David R. Maestas, Trin Murphy, Katarina M. Martinet, Tracey Moyston, Leon Xuanyu Min, Ali Behrangzade, Brock J. Pemberton, Sang-Ho Ye, George S. Hussey, Mohamad Azhar, William R. Wagner, Jonathan P. Vande Geest

**Affiliations:** 1 Department of Bioengineering, University of Pittsburgh, Pittsburgh, PA, USA; 2 Department of Medicine, University of Pittsburgh, Pittsburgh, PA, USA; 3 Department of Surgery, University of Pittsburgh, Pittsburgh, PA, USA; 4 McGowan Institute of Regenerative Medicine, University of Pittsburgh, Pittsburgh, PA, USA; 5 Department of Cell Biology & Anatomy, School of Medicine, University of South Carolina, Columbia, South Carolina, USA; 6 Department of Biomedical Engineering Program, University of South Carolina, Columbia, South Carolina, USA; 7 Department of Chemical and Petroleum Engineering, University of Pittsburgh, Pittsburgh, PA, USA; 8 Department of Mechanical Engineering and Materials Science, University of Pittsburgh, Pittsburgh, PA, USA; 9 Vascular Medicine Institute, University of Pittsburgh, Pittsburgh, PA, USA

## Abstract

Tissue-engineered vascular grafts (TEVGs) are promising alternatives for blood vessel replacement, yet their clinical translation is constrained by resource-intensive implantation studies. While necessary, implantation models obscure processes that govern long-term outcomes, such as time-resolved microscale remodeling. Here, we develop an organotypic culture that couples rat aortic explants with acellular grafts in a cylindrical geometry. Harnessing label-free non-destructive multiphoton imaging, we longitudinally assess fibrillar collagen via second harmonic generation (SHG) and simultaneously detect cellular and TEVG states by their respective two-photon excited fluorescence (2PEF). Fiber quantification unveils an evolving collagen architecture that is verified by orthogonal staining. We verify compatibility with common biomaterials, murine fluorescent reporters, and terminal assays. We demonstrate the platform’s perturbation sensitivity via treatment with transforming growth factor-β isoforms (TGF-β1/β2/β3) and identify shifts in fiber distributions coinciding with differential gene expression. The architectural fiber distributions observed in culture show agreement with 6-month interpositional aortic explants across two graft designs, supporting a trajectory-matching readout of remodeling. Collectively, this platform provides an accessible decision-informing tool to assess biomaterial scaffold designs prior to surgical implantation.

## Introduction

Cardiovascular disease remains the leading cause of global mortality, presenting a persistent clinical need for small diameter vascular replacements when autologous conduits are unavailable or insufficient^1–3^. Tissue engineered vascular grafts (TEVGs) have long been explored as promising alternatives, yet their clinical translation remains limited due to the complex host-graft integration processes that are difficult to interrogate during development^4–6^. Early interface events such as cellularization, extracellular matrix (ECM) deposition, and the emergence of fibrillar collagen architectures dynamically unfold to steer graft remodeling towards durable integration or towards failure modes such as stenosis or maladaptive fibrosis^4–7^. As a result, animal implantation studies remain essential for early TEVG design development, but these are resource-intensive, largely dominated by endpoint readouts and longitudinal ultrasound. As such, remodeling trajectories and spatial heterogeneity at the host-graft interface from these models are obscured. TEVG designs that pass initial mechanical characterization standards often proceed to implantation studies in small animals, and if successful, must be resized for large-animal long-term preclinical studies. Reflecting this developmental burden, meta-analyses have found that only a relatively small number of TEVG publications include *in vivo* implantation outcomes, and among all *in vivo* studies using acellular TEVGs, rodent models encompassed 59% of these reports, consisting primarily of mouse and rat interpositional implant models. Collectively, this suggests that a research translational bottleneck exists between TEVG design-to-benchtop characterization and successful animal implantation trials, wherein effective intermediate *ex vivo* models could reduce animal implantation failures and de-risk designs before commitment to resource-intensive studies^8–10^.

Culture systems that accurately correlate to *in vivo* studies are notoriously difficult to achieve, though recent breakthroughs in three-dimensional (3D) cultures and tissue explant *ex vivo* systems have reported outcomes that are superior or comparable to monolayer cultures^11–13^. Conventional TEVG *in vitro* models span simple monolayer cultures for high-throughput, but poorly representative microenvironments, to sophisticated modern vascular bioreactors capable of providing luminal pulsatile flow and tunable mechanical stimuli during culture^14–16^. While these advanced models provide valuable insights, they are often costly, complex, reliant on coarse longitudinal readouts, and rely heavily on terminal endpoint assays to assess integration. Across these approaches, a persistent limitation is the lack of non-destructive, high-resolution, longitudinal readouts that can quantify ECM remodeling and cellularization at the tissue-graft interface where failure modes are often concentrated. While recent organotypic and explant-based vascular culture systems have begun to close aspects of this gap, these are largely configured to culture blood vessels or TEVGs separately, focused on short-term hemocompatibility, brief in duration (2 weeks), or monitored using macroscopic imaging modalities that do not resolve evolving collagen architecture^17–24^. As a result, there remains a need for an accessible intermediate model that maintains geometry-preserving cylindrical artery-graft coupling while enabling repeated, label-free, microscopic assessment of ECM remodeling trajectories across multiple time ranges.

The purpose of this work is the development of a three-dimensional (3D) organotypic artery-graft interface culture platform to serve as a bridge between monolayer culture assays and rodent implantation to improve graft screening and design decision factors. In our platform, we directly couple rat (or mouse) aortic explants to acellular TEVGs (or autologous grafts) to preserve a biomimetic cylindrical tissue-graft geometry *ex vivo*. We pair this culture approach with label-free, non-destructive multiphoton imaging to repeatedly monitor fibrillar collagen deposition and remodeling via second harmonic generation (SHG) alongside endogenous two-photon excited fluorescence (2PEF) signatures of cellularization and scaffold-associated background signals utilizing a single excitation-collection scheme^25,26^. Using fiber-level quantification of SHG images, we capture evolving collagen architecture across culture duration, and we validate cellular 2PEF signatures using live and fixed nuclear staining, and we confirm the platform’s compatibility with standard terminal assays including gene expression, histology, and immunostaining^27–29^. We explore the potential for studying aortic-injury model contexts, platform compatibility with murine and fluorescent reporter models, and we confirm that various biomaterial-based grafts are compatible with longitudinal tracking except in those with intrinsically high SHG signatures. We compare the culture-derived collagen architecture to long-term explant outcomes across two distinct TEVG designs and find that they share multiple similarities in fiber metrics. Finally, we demonstrate the platform’s sensitivity to biochemical perturbations and find that stimulation with transforming growth factor-β isoforms elicits distinct collagen gene expression responses coinciding with measurable shifts in SHG-derived fiber architecture distributions^30,31^.

## Results

### Design of a 3D artery-TEVG *ex vivo* culture with longitudinal label-free readouts

**Fig. 1 |:**
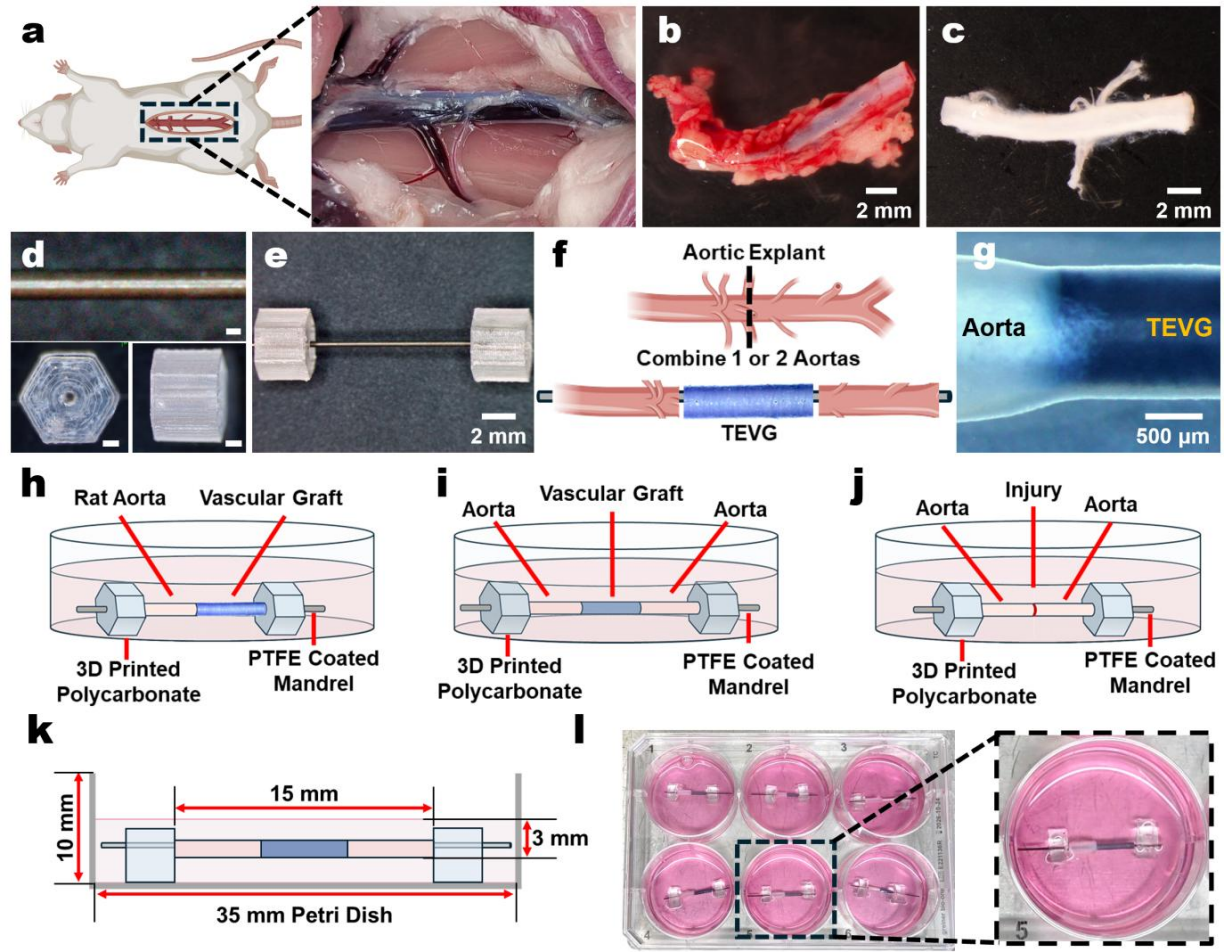
Design of the 3D TEVG-Artery *Ex Vivo* Culture. **a-c.** Visualization of rat aortic isolation and preparation by removal of peri-adipose tissue. **d.** Assembly parts of the 3D culture (top) PTFE coated stainless steel mandrel and (bottom) 3D printed polycarbonate hexagonal sample holders. **e.** Necessary parts and assembly of the 3D culture model. **f.** A graphical illustration of a double cannulated TEVG to mimic implantation. **g.** Representative zoom-in of the tissue-TEVG interface. **h-j.** Illustrations of tested 3D aortic culture variations depicting (**h**) a single connection, (**i**) an interpositional (double) connection, and (**j**) a transected and rejoined aorta to model aortic injury and/or ideal autologous grafts. **k.** Schematic showing culture dimensions. **l.** Image of a 6-well plate of single joined cultures. Scale bars in d are 1 mm.

We first established baseline multiphoton signatures for native rat aorta to define interpretable label-free channels for fibrillar collagen and tissue autofluorescence (Extended Data Fig. 1a). Mounted aortic cross-sections and fresh explants produced a robust SHG signal and endogenous 2PEF under 792 nm excitation, with strong emission in the 460/40 nm window and weaker signal in the 620/60 nm window (Extended Data Fig. 1b). To confirm that the system could report remodeling over time, we cultured diced aortic explants and observed the emergence of cell-associated 2PEF by 2 weeks, followed by prominent SHG-positive fibrillar structures by 4 weeks (Extended Data Fig. 1c,d). Building on these signatures, we developed a modular organotypic artery-TEVG interface culture to preserve cylindrical geometry while remaining compatible with standard culture formats. Rat abdominal aortas were harvested, trimmed, and cleaned of perivascular tissue, then supported on PTFE-coated mandrels using sterilizable 3D-printed polycarbonate holders to elevate the construct above the dish surface (Fig. 1a–e). The aorta was transected and coupled to an acellular graft scaffold to form either a single-ended interface configuration for tracking cellular ingress or an interpositional (double-ended) configuration that better approximates *in vivo* implantation geometry (Fig. 1f–j). Of particular interest was that the platform could also allow us to model aortic injuries or autologous graft controls (Extended Data Fig. 2). Across configurations, approximate diameter matching between the graft and host vessel minimized telescoping and improved longitudinal monitoring of cellularization and remodeling (Extended Data Fig. 3, Fig. 1g). The assembled construct sits within 35 mm dishes and 6-well plates, enabling parallelized testing without specialized bioreactor hardware (Fig. 1k,l).

### Scaffold two-photon autofluorescence can be mapped to support longitudinal segmentation strategies

**Fig. 2 |:**
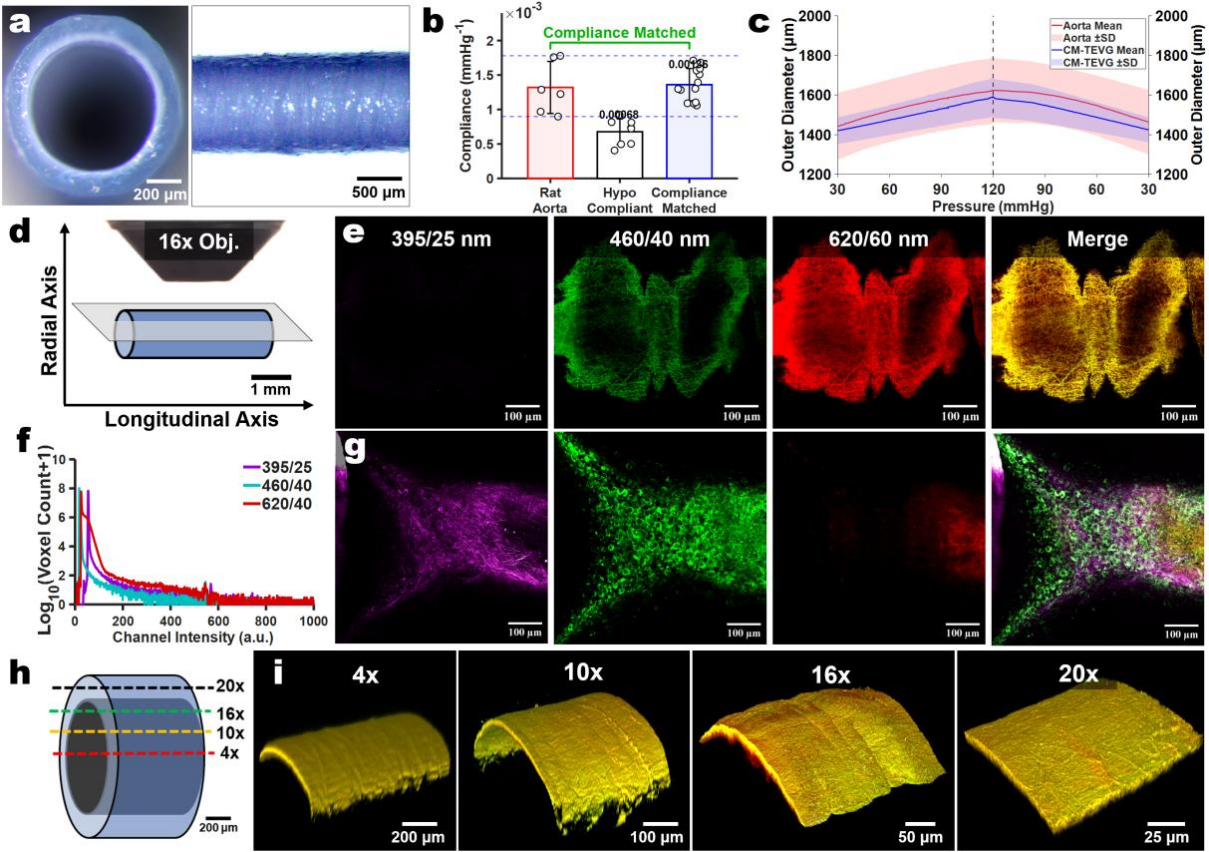
Mechanical compliance matching and multiphoton spectral signature of TEVGs. **a.** Representative views of the acellular trilayered TEVG used for imaging/background characterization, shown in cross-section (left) and longitudinal view (right). **b.** Compliance comparison between native rat abdominal aorta, a hypocompliant TEVG (comprised of a thicker middle layer), and a compliance-matched TEVG (n = 6–10). **c.** Pressure-outer diameter response (mean ± s.d.) for rat aorta versus the compliance-matched TEVG, illustrating diameter distension behavior across physiological pressures. **d.** Imaging orientation schematic used for image acquisition. **e.** Representative scaffold-only multiphoton images acquired under 792 nm excitation in the three collection windows used throughout this study: 395/25 nm (SHG), 460/40 nm (2PEF/endogenous), and 620/60 nm (2PEF/autofluorescence), with merged view shown at right. **f.** Histogram overlays of voxel-intensity distributions from a scaffold-only 3D stack (log-scaled voxel count), defining the relative background signature in each channel under the same settings used for live longitudinal imaging. **g.** A representative 8-week culture with aortas and respective signals in each of the three channels. **h.** Illustration of typical depth penetration/working depth achievable in the configuration across common objective magnifications (4x, 10x, 16x, 20x). **i.** 3D reconstructions of the merged scaffold background signal at each magnification to show the volumetric appearance of the scaffold in the absence of tissue/cells. Scale bars in (i) are relative approximations for scale reference.

To support implant-relevant benchmarking of the culture platform’s remodeling trajectories, we anchored our imaging characterization to a compliance-matched trilayered TEVG that was used in parallel with a companion study on long-term rat interpositional implantation remodeling. Our baseline grafts are comprised of electrospun polyester urethane urea (PEUU) mixtures with porcine gelatin for the outer layers, and a zwitterionic sulfobetaine-modified variant of PEUU (PESBUU-50) for the luminal layer to support hemocompatibility^32,33^. The TEVGs were crosslinked with genipin to stabilize the gelatin content, resulting in a blue coloration (Fig. 2a). We iteratively tuned the layer thicknesses to match circumferential compliance of native rat abdominal aorta under closed-end tubular biaxial testing at physiologically relevant conditions. Using outer diameter (OD) tracking during sinusoidal pressurization, we calculated (70–120 mmHg) circumferential compliance (Fig. 2b,c). We standardized our imaging workflow using a fixed acquisition geometry (16x water-dipping objective positioned over a TEVG mounted in a 35 mm dish) to ensure stable immersion and reproducible spatial sampling across timepoints (Fig. 2d). Under 792 nm excitation, TEVG-only signatures exhibited modest signal in the 460/40 nm channel, robust signal in the 620/60 nm channel, and minimal-to-no SHG signal in the 395/25 nm channel, enabling clear separation of scaffold-associated background from collagen-associated SHG (Fig. 2e–f). To assess the depth limitations when using backward (epifluorescent) signal collection, we evaluated several common two-photon objectives (4x, 10x, 16x, & 20x). Across these, we found a consistent 3D structural signature that can be leveraged as an internal reference for longitudinal registration and segmentation during image post-processing (Fig. 2h–i). As various biomaterials and processing techniques can yield differing 2PEF signatures, this motivated us to assess the generalizability of our platform. We applied the culture method across several TEVG biomaterial classes (electrospun gelatin, PEUU, PESBUU-50, PCL, and UBM-hydrogel coated constructs) in 4-week culture studies and found that each supported consistent longitudinal imaging, and we identified scaffold specific differences, underscoring how biomaterial selection may be critical factor in the subsequent collagen remodeling outcomes upon vascular grafts (Extended Data Fig. 4 & 5)^34^.

### Multiphoton imaging enables 4D tracking of collagen-associated signatures

**Fig. 3 |:**
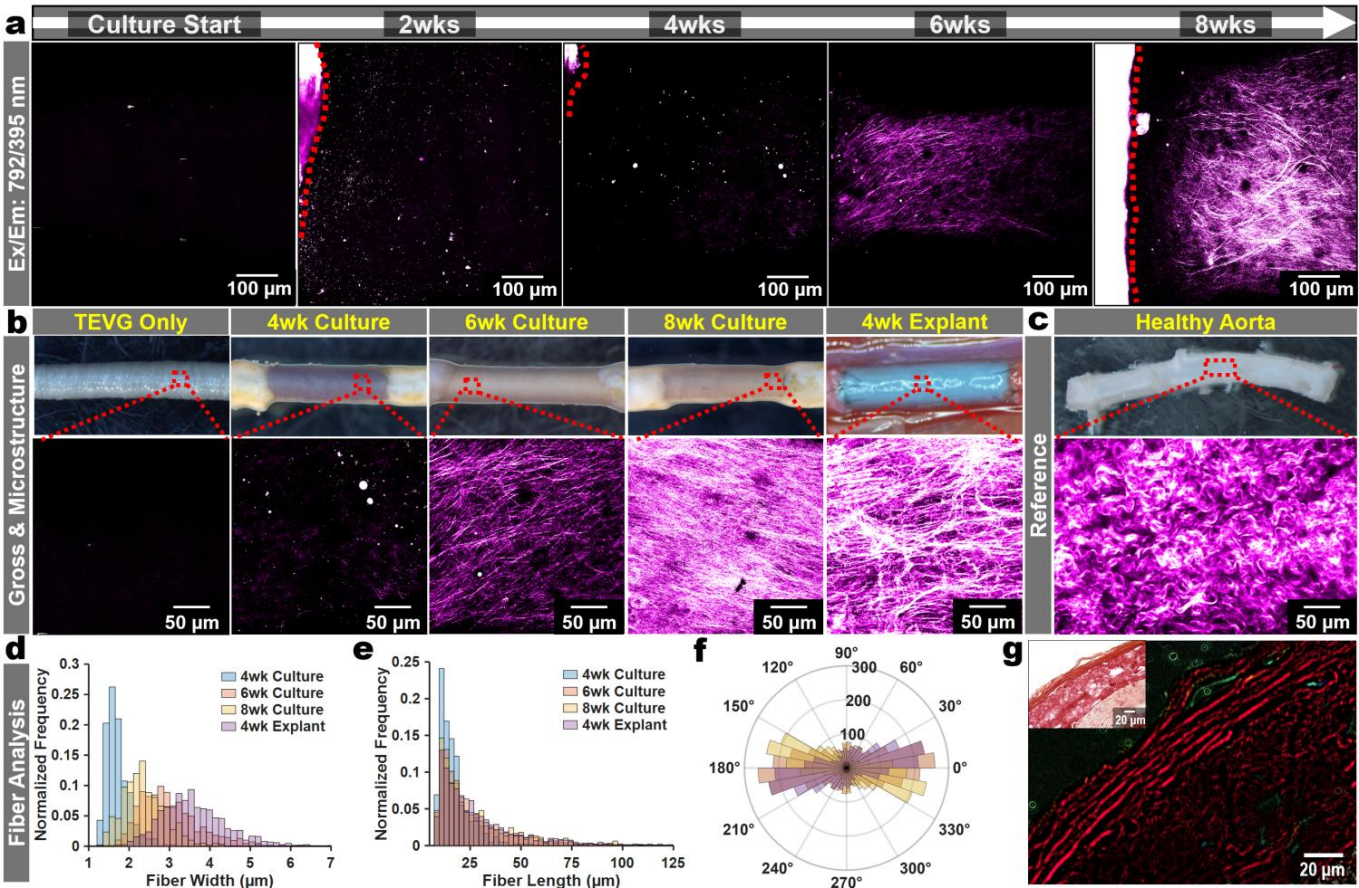
Multiphoton Longitudinal SHG Assessments. **a.** A representative series of time course images of 395/25 nm emission after excitation of 792nm to yield SHG signatures from the culture start through 8-weeks of culture. Red dashed lines indicate where aorta is in view. **b**. Representative images of the TEVG only, TEVGs cultured for 4wks, 6wks, & 8wks, and the same design graft implanted as an interpositional aortic graft at 4wks. **c**. A healthy aorta’s SHG for reference. **d.** SHG fiber analysis using CT-FIRE Fiber width normalized to each sample’s (from b) total fiber counts **e**. fiber length, and **f.** fiber angles. **g**. 8-week cultured TEVGs sectioned and stained with picrosirius red to visualize collagen. The top left panel shows the gross picrosirius red staining (60x objective) and the larger panel depicts polarized light imaging to depict collagen birefringence.

We sought to longitudinally track SHG upon the graft surfaces across culture duration. We found that SHG was trackable using a 16x (NA = 0.8) objective, and to a limited degree with a low-NA 0.28 4x objective, which may suggest that the system can be adapted for rapid, coarse screening of remodeling over larger fields of view prior to higher-resolution acquisition (Extended Data Fig. 6). As we compared our multiphoton imaging to conventional methods, we found that the platform supports, to a limited extent, monitoring using commonly available imaging modalities (surgical scope/reflectance, inverted phase contrast/brightfield, and upright epifluorescence), enabling low-barrier routine inspection of tissue-scaffold contact and gross remodeling (Extended Data Fig. 7). When the model is paired with multiphoton microscopy this enables repeated, non-destructive assessment of fibrillar collagen upon the same constructs. In time-course imaging datasets, we found a progressive intensification of fibrillar SHG-positive signal (Fig. 3a). We then quantified collagen architecture using an established open-source SHG-fiber analysis pipeline (CT-FIRE), allowing us to characterize microstructural features such as SHG-fiber width, length, and straightness. This provided us with the ability to track microstructural readouts of ECM remodeling from SHG signatures across time, appearing in baseline artery-TEVG cultures by weeks 2 as short microfibers, and then as mature fibers after 4 weeks (Fig. 3b–e). In agreement with prior reports, we found that the SHG-based fiber metrics exhibited modest sensitivity to sample orientation during imaging, motivating us to perform all acquisitions in a standardized orientation across studies (Extended Data Fig. 8)^35,36^. Finally, collagen deposition was corroborated by picrosirius red staining (Fig. 3f–g).

### TEVG cellularization tracking and validation of cell-associated 2PEF corresponding to nucleated cells

**Fig. 4 |:**
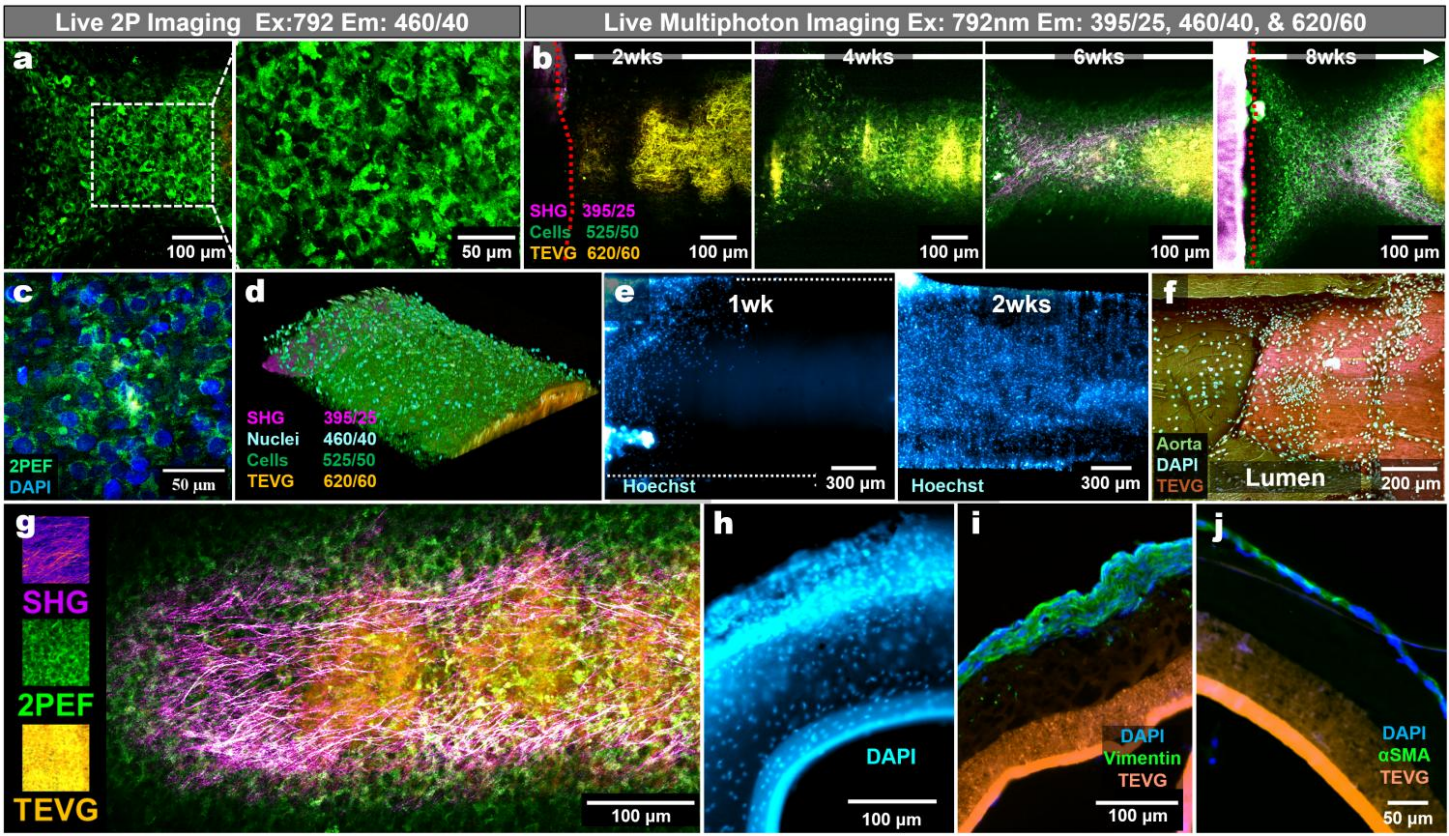
3D Culture Cellularization and Longitudinal Tracking. **a.** Representative 20x image of the 460 nm 2PEF channel (Ex/Em: 792/460) highlighting cell-like structures developing within/upon the TEVG surface. **b.** Longitudinal live two-photon imaging of TEVG cellularization in the 3D *ex vivo* culture over 2–8 weeks. Images are displayed as merged channels under 792 nm excitation, showing SHG (magenta), cellular two-photon excited fluorescence (2PEF; green), and TEVG background/autofluorescence (red) wherein overlapping red and green appear as yellow/orange. **c.** Merged before and after images to verify that 460 nm 2PEF corresponds to nucleated cells (DAPI). **d.** A fixed and DAPI stained sample was cut, and the inner lumen was imaged facing upward. 2PEF from 720 nm excitation shows the aorta (dark green, 460 nm), the TEVG (orange, 395 nm), and DAPI-stained nuclei (cyan, 525 nm). **e.** Validation of cellular presence and compatibility with standard microscopy: widefield inverted fluorescence images of Hoechst-stained cultures at 1 and 2 weeks to demonstrate surface cell tracking. **f.** A 3D reconstructed view of Hoechst-stained nuclei across the TEVG after 2 weeks of culture. **g.** A representative high-resolution image at 16x showing an 8-week culture sample, able to distinguish cells, SHG, and the TEVG. The aorta is off-screen, to the left. **h.** Gross cross-section of a 2wk cultured TEVG (near the aortic interface) fixed and stained with DAPI to visualize nuclei. **i.** Immunostaining for vimentin. **j.** Immunostaining for α-SMA. Red dashed lines (b) indicate areas where the aorta is in view.

Longitudinal live multiphoton imaging revealed progressive emergence of cell-associated 2PEF on the TEVG surface and at the aorta-TEVG interface over 2–8 weeks of culture (Fig. 4a). To verify that elevated 460-channel signal represented cellular structures rather than scaffold autofluorescence, fixed constructs were imaged, then stained with DAPI, and re-imaged. Regions with strong 460 signals colocalized with nucleated cells (Fig. 4b). Post-fixation imaging also enabled visualization of cellular infiltration along the luminal aspect of the interface region after transecting the sample (Fig. 4c). Endogenous cell-associated 2PEF was weak at early time points, so we verified that live Hoechst staining provided a complementary approach to track nuclei migration across the TEVG beginning at 1–2 weeks (Fig. 4e–f). High-resolution imaging distinguished cell-associated 2PEF, SHG-positive collagen, and TEVG-associated autofluorescence (Fig. 4g). We verified that cells were infiltrating the TEVG at the interface and used immunofluorescent staining to assess general cell phenotypes near the middle of the TEVGs (~4 mm from the aorta) and found expected signatures for vimentin and alpha-smooth muscle actin (α-SMA) (Fig 4h–j). Few genetically modified fluorescent reporters exist for rat models, motivating us to determine whether our platform was compatible with murine aortic explants, including a murine dual-reporter relevant to vascular researchers (Myh11-CreERT2;Rosa26-mTmG). We verified that our platform was compatible with a tamoxifen induced smooth muscle lineage-resolved tracking reporter and enabled wavelength-tunable reporter visualization during live multiphoton imaging (Extended Data Fig. 9).

### Remodeling trajectories in the culture model mirror long-term remodeling *in vivo*

**Fig. 5 |:**
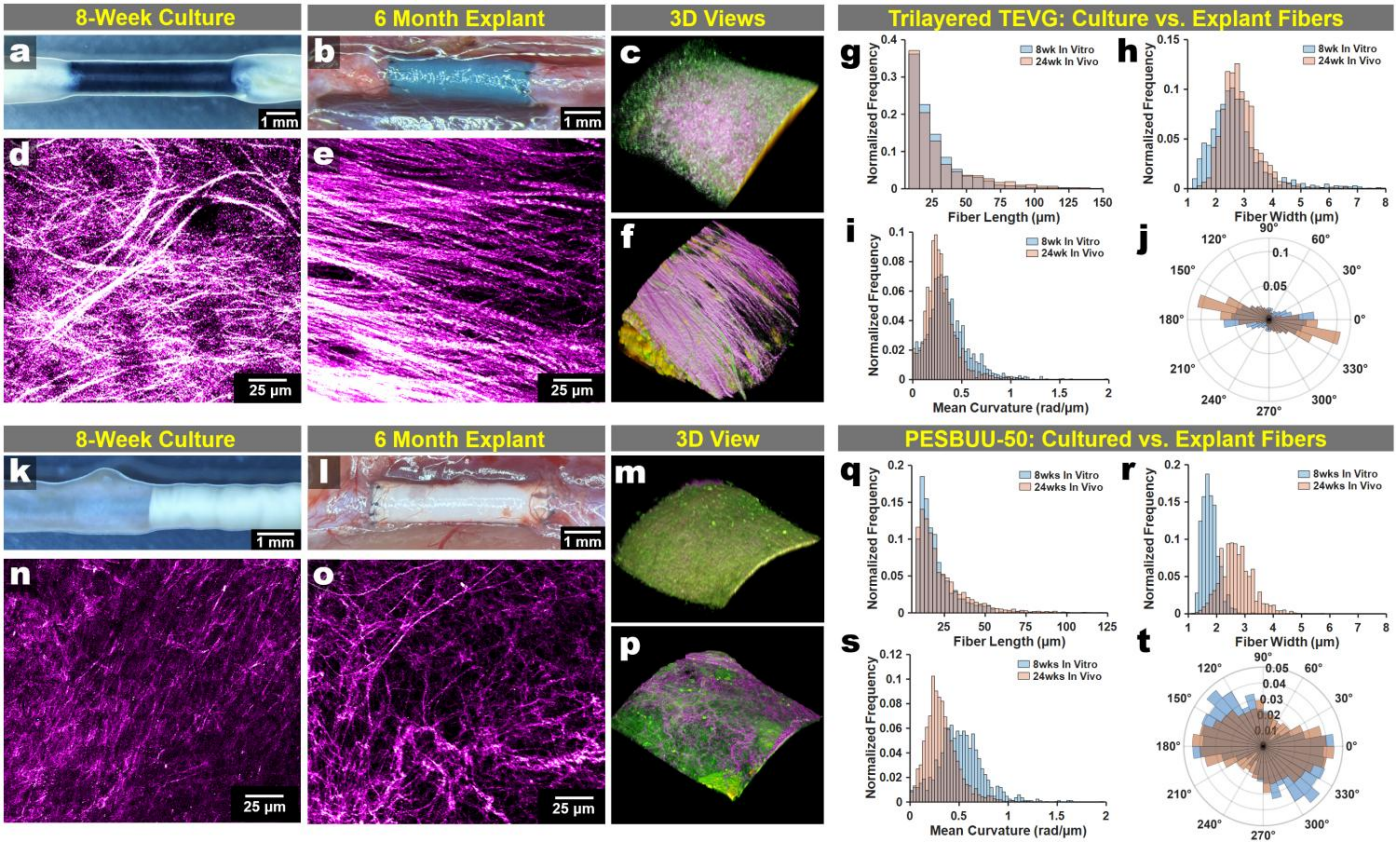
*Ex vivo* culture recapitulates key collagen microstructural features observed after implantation across two TEVG designs. Trilayered TEVG: **a,** Representative gross images of an 8-week cultured graft. **b.** The same graft design in situ at 6 months post-implantation immediately prior to explant. **c.** Representative 3D projection of the 8-week culture. **d,e.** Second harmonic generation (SHG) maximum intensity projections (MIPs; magenta hot) from 8-week culture (**d**) and 6-month explant (**e**). **g-j.** Collagen fiber metrics quantified from SHG images (blue, 8 weeks *in vitro*; orange, 24 weeks *in vivo*): (**g**) fiber length, (**h**) fiber width, (**i**) mean curvature (rad/μm), and (**j**) fiber orientation. **PESBUU-50 TEVG:** (**k,l**) Representative gross images of an 8-week cultured graft (**k)** and 6-month explant *in situ* (**l**). **m.** A 3D reconstructed view of the 8-week culture. **n,o.** Representative SHG MIPs from 8-week culture (**n**) and 6-month explant (**o**). **p.** A 3D reconstructed view of the explanted graft. (**q-t**) Corresponding SHG-derived fiber metric distributions (blue, 8 weeks *in vitro*; orange, 24 weeks *in vivo*): (**q**) fiber length, (**r**) fiber width, (**s**) mean curvature, and (**t**) fiber orientation.

We then investigated whether the longitudinal remodeling signatures captured in the organotypic culture reflected the microstructural remodeling observed in our long-term implantation studies using the same graft designs. We utilized a rat lower abdominal interpositional implant model using the compliance-matched trilayered TEVGs for 6 months. In parallel, utilized the same TEVGs in our culture model and acquired SHG signatures from both at matched en face orientations (Fig. 5a–f). We found that SHG-derived fibrillar collagen architecture in the culture exhibited agreement with the dense, aligned fiber phenotypes observed in 6-month explants (Fig. 5c,f). We quantified collagen fiber organization using SHG-derived metrics (fiber length, width, curvature distributions, and angular distributions) as before, comparing the cultures against the 6-month models (Fig. 5g–j). In trilayered grafts, distributional comparisons demonstrated overlapping profiles across multiple fiber descriptors. We also compared our 8-week culture results with 6-month studies using an electrospun PESBUU-50 TEVG design. We found that SHG phenotypes at 8 weeks in culture qualitatively resembled those in 6-month explants (Fig. 5k–p), with differences in fiber width and curvature in SHG-derived fiber metric distributions (Fig. 5q–t).

### The 3D culture model is sensitive to exogenous protein treatments

**Fig. 6 |:**
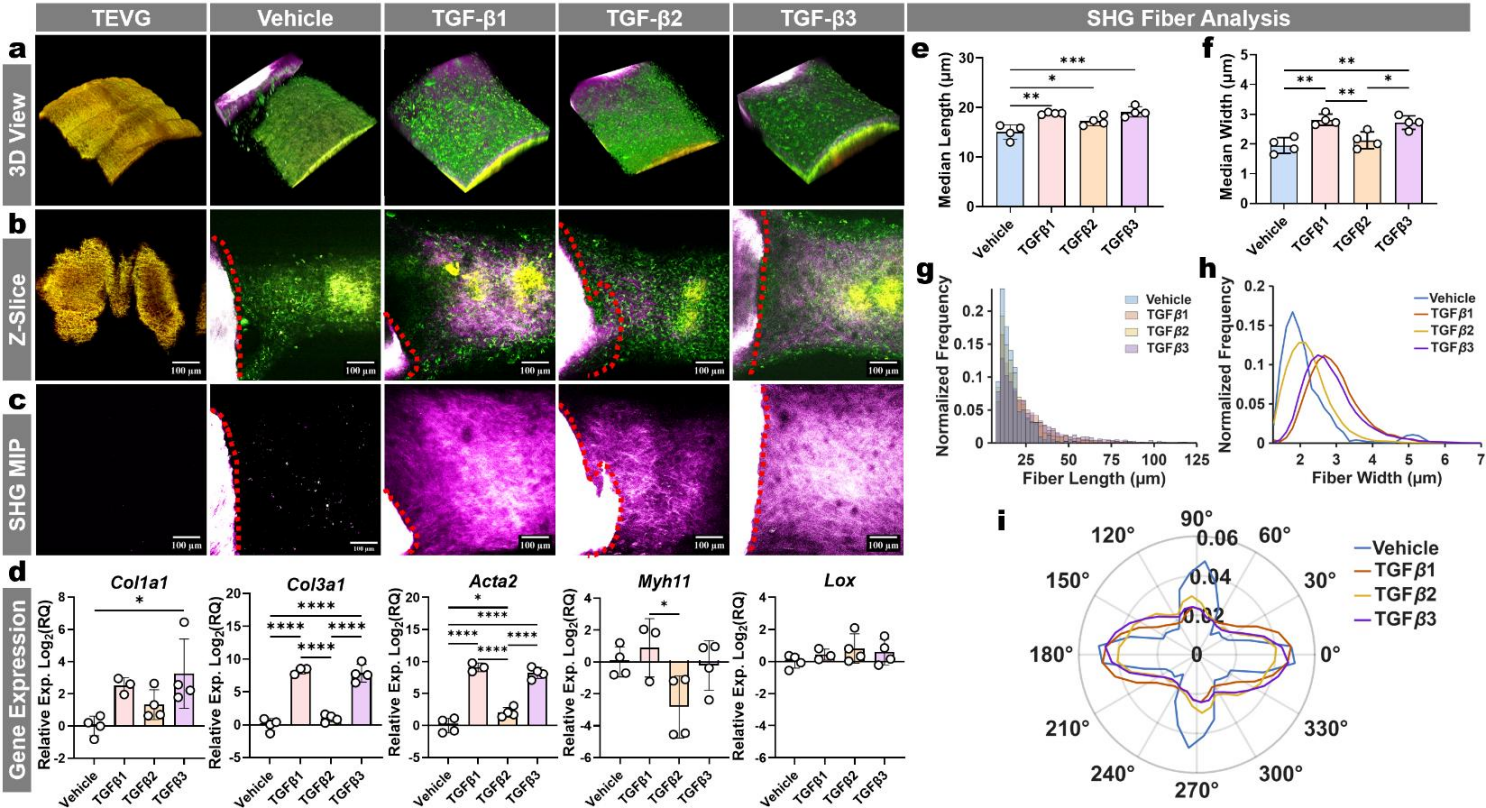
3D Culture Sensitivity to Exogenous TGF-β Isoform Treatments. a. Representative 3D reconstruction of a 4wk cultured sample from each group. **b.** Representative z-slice images of 4wk cultured samples stimulated with 10ng/mL of rhTGF-β isoforms every three days. **c.** A representative SHG MIP shown from each group. **d.** Gene expression levels of the rhTGF-β treated groups normalized to the vehicle treated group. Reference gene was *Rer1*, and results depicted on a Log_2_(Fold-Change) scale. **e.** Collagen fibers were segmented from SHG (395/25 nm) channel MIPs using CT-FIRE, and fiber length and **f.** fiber width were quantified for each replicate. For each replicate, the median fiber length (**e**) and median fiber width (**f**) were calculated across all detected fibers, and these replicate medians were used for plotting and statistics. **g,h.** Plots of the normalized frequency of each group’s fiber lengths and widths (each replicate normalized to its respective total fibers). **i**. Polar plot shows the normalized distribution of collagen fiber orientation angles for each group. Fiber angles were binned (10° bins), replicate histograms were normalized to probability, and the group trace represents the mean across replicates. Statistics (qPCR) were performed on each gene using ordinary one-way ANOVA with Tukey post-hoc (n = 4). Statistics for (e) and (f) are ordinary One-way ANOVA followed by a Tukey post-hoc test (n = 4). *P≤0.05, **P≤0.01, ***P≤0.001, and ****P≤0.0001.

To demonstrate the system’s responsiveness, we evaluated whether SHG was impacted by human recombinant TGF-β isoform treatment, well-established inducers of collagen deposition in aortic culture contexts. Longitudinal tracking of each condition (n = 4 independent cultures per group) showed increases in SHG intensity at 4-weeks with TGF-β isoforms 1, 2, and 3 treatments (10 ng/mL every 3 days) relative to vehicle controls. Across 3D reconstructions, z-planes, and max intensity projections of SHG, we found a strong increase in SHG intensity and appearance with TGF-β isoform treatment (Fig. 6a–c). To validate these results and verify that the culture system remains compatible with endpoint assays often used in vascular contexts, we performed qRT-PCR at 4 weeks and found that TGF-β isoforms altered expression of contractile and matrix-remodeling markers (including *Myh11*, *Acta2, Lox, Col1a1*, and *Col3a1*) relative to vehicle controls, supporting that the observed microstructural SHG differences are accompanied by significant differences in gene expression (Fig. 6d). We next quantified SHG fiber architecture using CT-FIRE. In comparison to vehicle treatment, TGF-β treatment shifted fiber-length and fiber-width distributions and altered group medians, demonstrating that the platform resolves remodeling at the level of collagen microstructure rather than relying solely on qualitative imaging (Fig. 6e–h). Notably, TGF-β2 produced narrower fibers than TGF-β1 and TGF-β3, consistent with its more modest induction of collagen-associated readouts (Fig. 6f,h). In contrast, fiber orientation distributions were broadly similar across treatment groups, suggesting that TGF-β primarily modulated fiber size/scale under these conditions rather than imposing a dominant alignment axis (Fig. 5i). We further assessed whether these trajectories were maintained after ceasing isoform treatment by 4 weeks and found that TGF-β1 and -β3 groups had substantial increases in SHG signal by 8 weeks (4 weeks after ceasing isoform treatments), while the TGF-β2 treated group maintained qualitatively thinner fibers and lower SHG intensity (Extended Data Fig. 10).

## Discussion

A persistent bottleneck in small-diameter vascular graft development is the requirement for surgical implantation models to assess graft performance, yet these studies are time and resource intensive. While small and large animal implantation models remain the preclinical gold standard, these intrinsically limit iteration across scaffold designs, bioactive modifications, and perturbations, ultimately slowing development. Here, we introduce a geometry preserving organotypic artery-graft culture model that enables longitudinal, label-free multiphoton readouts of tissue-graft integration across multiple weeks of culture. This method maintains a 3D cylindrical tissue-TEVG interface, approximating that of interpositional grafting, and uses standard culture plastics and purchasable materials. This design enables optical access to the top and bottom for microstructural, interface-resolved imaging to assess cellular infiltration and fibrillar matrix remodeling. By supporting the explant and scaffold on PTFE coated mandrels and 3D printable height-matched holders, the system stabilizes tissue-graft apposition without sutures, adhesives, or clamps, allowing those clinically relevant factors to be introduced as deliberate experimental variables rather than as covariates. Due to this methodology, we found that dimensional matching between the host vessel and scaffold strongly shaped interface behavior. This sensitivity to geometry is consistent with recent reports on the impact of tissue-graft dimensional mismatches and their role in patency, further supporting that graft failure can emerge from wall remodeling programs and anastomotic mechanics^37^.

Using this platform, we demonstrate the ability to track fibrillar collagen remodeling (SHG) and endogenous cell-associated signals (2PEF) as they evolve on the TEVGs over time. This methodology thus provides an intermediate capability previously lacking in TEVG research between benchtop TEVG development and animal implantation and provides performance outcomes for graft remodeling and cellularization in a physiologically relevant microenvironment. As we assessed fibrillar collagen development, we found that the platform’s multiphoton readouts were compatible with established SHG fiber-extraction tools (CT-FIRE) to provide quantitative metrics such as fiber length, width, and orientation distributions. We further utilize picrosirius red staining to support that the label-free SHG readouts correspond to collagen-rich remodeling features. The label-free 2PEF signal was spatially cell-associated, with punctate/perinuclear fluorescence patterns and nuclear exclusion that co-registered with DAPI-positive nuclei in endpoint images, supporting interpretation of the signal as a cellular autofluorescence readout. We further demonstrate that live nuclear dyes, such as Hoechst, can provide a practical complementary strategy for tracking cell migration across the grafts and for use at early time points to identify cells when endogenous 2PEF signatures are weak.

Using the platform’s ability to track collagen remodeling and cellular presence, we evaluated its sensitivity in detecting differences arising from controlled biochemical perturbations relevant to vascular matrix remodeling. Using TGF-β isoforms due to their general usage as canonical drivers of collagen deposition and remodeling in vascular contexts, we found that isoform treatments produced dramatically higher increases in SHG signal relative to vehicle conditions, indicating that the platform is responsive to biologically meaningful stimuli. Importantly, the system remains compatible with downstream assays: qRT-PCR performed at four weeks captured coordinated changes in contractile and matrix-remodeling markers (including *Myh11, Acta2, Lox, Col1a1,* and *Col3a1*), supporting that structural differences observed by SHG correspond to transcriptional remodeling programs. Fiber-level quantification further suggested that TGF-β isoforms can produce distinguishable microstructural patterns (e.g., differences in fiber width).

Intrinsically, off-the-shelf TEVGs must be designed to account for the resulting biomechanics, biochemistry, adsorption, and toxicity of each design. This poses a strong need for biocompatibility screening methods to vet potential designs, yet with assays that have tolerance for diverse scaffold compositions. We therefore tested multiple TEVG-relevant biomaterials for compatibility with our system and observed that these can be monitored using the same acquisition settings, and we find preliminary evidence that each biomaterial choice may impact the resulting SHG and cellular 2PEF outcomes. In agreement with prior reports, we observed that pepsin-digested UBM hydrogel coatings retained residual SHG, which limited our ability to monitor *de novo* SHG, but we found that these could still be assessed for cellularization via endogenous 2PEF. This suggests some natural biomaterials may require additional processing or methodological variations to track *de novo* SHG with this platform. To further assess broader applicability of this platform, we cultured murine and genetically encoded fluorescent reporter models. Using murine aortic explants and a dual reporter model (GFP Myh11 lineage reporter with TdTomato Red for all others), we show that reporter tissues can be integrated into the artery-TEVG culture to capture SHG remodeling and cellularization at our standardized excitation/emission settings, and that the reporters can still be isolated under their respective excitation wavelengths. This capability expands potential applications relevant to vascular and aortopathy research by utilizing transgenic reporter strains such as ROSA26-mTmG, BPAN, and AngII for studies investigating how single-cell populations contribute to early interface remodeling. We also found that this system could be used to track arterial repair and rejoining after injury, potentially introducing a new model to assess the conditions that drive healthy remodeling in contrast to maladaptive vascular scarring, foreign body responses, and their respective mechanistic underpinnings.

While versatile, this platform has several limitations that define its intended use. Our baseline configuration does not include known drivers of vascular remodeling, such as hemodynamic flow, mechanical loading, and the role of endothelial cells. Mirroring the aortic ring assay, we utilize a static design positioned near the media interface to allow sufficient gas perfusion. While modern bioreactors can provide luminal perfusion and mechanical stimulus, each of these additions can escalate the likelihood of failure modes and decreases the number of replicates that can be run in parallel. As with all *ex vivo* systems, our model lacks systemic contributors from circulating blood components, such as various infiltrating immune and progenitor populations, innervation, and multi-organ signaling. As such, our platform’s optimal use is to inform upon graft design decisions prior to, or in parallel with, animal implantations. Although we demonstrate its performance as a trajectory mapping model, it is not a surrogate for *in vivo* studies. Further, this platform uses SHG-fibrillar signals as a proxy measure of collagen deposition and remodeling. While multiphoton acquired SHG is considered a gold standard for identifying the presence of fibrillar collagen, it does not measure total collagen content directly and can underrepresent disordered, very thin, or non-fibrillar collagen features. Acquisition choices and fibril architecture can influence SHG intensity and fiber readouts; thus, we recommend that SHG outcomes in this model to be treated as a semi-quantitative proxy of collagen organization and fibrillar structure. Studies that seek definitive total collagen quantification and compositional remodeling should be complemented by terminal orthogonal biochemical assays.

Collectively, this artery-TEVG culture and imaging platform provides an accessible, throughput-scalable intermediate platform for interrogating early host-graft interface remodeling with longitudinal, label-free multiphoton readouts. By combining its facile design, repeated microstructural monitoring, compatibility with molecular endpoints, and feasibility with murine reporters, this method creates a practical bridge between monolayer *in vitro* assays and resource-intensive implantation studies. Positioned as a remodeling trajectory predictor and screening system, it has potential applications for mechanistic probing, hypothesis pruning, and the potential to accelerate both TEVG development and autologous arterial graft research by identifying remodeling trajectories and failure modes before committing to *in vivo* experimentation.

## Methods

### Materials and custom components

Reagents, consumables, equipment, and vendor catalog numbers are provided in Extended Data Table 1. Hexagon-shaped 3D-printed polycarbonate (PC) sample holders were used to provide consistent height alignment and stable tissue-TEVG contact and allow rotation of the samples, as needed. The hexagonal designs consisted of a 5 mm × 5 mm × 5 mm shape with a 0.5 mm hole at the center. 3D printing was performed using an Ultimaker Pro 5. Printing settings were set to an infill of 100% using transparent PC (Ultimaker, USA). This design is available as a downloadable .STL file.

After printing, PC holders were inspected and cleaned with sterile DI water, soaked in 70% EtOH for 4 h to remove any free unbound polymers, and then sterilized by autoclave at 121°C while on a 500 μm OD mandrel. Before use, the holders were placed in sterile 1x DPBS overnight and rinsed in sterile 1x DPBS immediately prior to use. PC holders are disposable, but reuse is feasible with sterilization. PTFE-coated stainless-steel wire mandrels with a 500 μm outer diameter (OD) were cut to 30 mm lengths and used to support and align the samples above the culture surface. Mandrels were sterilized by autoclaving at 121 °C. Mandrels were intentionally extended beyond the sample holders to enable handling with sterile forceps during assembly and transfer.

### Culture medium preparation

The presented culture system utilizes complete Smooth Muscle Cell Growth Medium (SmGM-2; Lonza, Germany). Complete media was prepared according to the manufacturer’s instructions and supplemented with the proprietary SingleQuots SmGM-2 vials which include 25 mL of FBS (5%), insulin, hFGF-B, hEGF, and GA-1000 (gentamicin sulfate-amphotericin). At the time of this publication, the vendor verified that SmGM-2 is based on MCDB 131 media containing 5.55 mM glucose, 1 mM pyruvate (pyruvic acid), and 10 mM glutamine. As MCDB 131 media was originally formulated to include trace elements, we confirmed with the vendor that SmGM-2 includes 0.005 μM cupric sulfate pentahydrate (1.25 μg/L). Complete media was aliquoted into sterile vials and stored at 4°C in a light-protected box and was used within 1 month of preparation. Before changing, aliquoted media was covered with a gas-permeable sterile membrane film and placed into a CO_2_ incubator (37°C, 5% CO_2_) for 30 mins prior to use to stabilize pH and temperature.

### Rat and mouse aortic explant isolation

All studies were performed under IACUC approved animal care protocols. Lower abdominal aortas were harvested from young adult male Sprague Dawley rats (6–8 weeks old, ~200 g). Prior to explant, animals were sprayed with 70% EtOH before entry into the biosafety cabinet. Using pre-sterilized tools, the abdomen was opened to expose the aorta, and organs were gently displaced to minimize rupture and bleeding. The aorta was clamped above the aortic bifurcation and below the infrarenal region to obtain ≥ 2 cm of vessel. To minimize exposure to air, the aorta and vena cava were explanted together and submerged in 4°C sterile 1x DPBS supplemented with 2% penicillin-streptomycin and 2% amphotericin B. These were separated under a stereomicroscope and gently flushed to remove any residual blood and clots. Aortas were handled consistently and cleared of peri-adipose tissue using sterile forceps, with care to minimize damage to the adventitia of the vessel. The total time from euthanasia to final placement in the incubator was maintained at ≤ 45mins per sample (average time ~30 mins). Longer processing times (>1 hr) led to reduced cell growth.

For murine wild-type explants, adult male C57BL/6J mice were purchased from Jackson Laboratories. For smooth muscle lineage tracing, B6.FVB-Tg(Myh11-icre/ERT2)1Soff/J mice (JAX Stock# 019079) were crossed with B6.129(Cg)-Gt(ROSA)26Sor^tm4(ACTB-tdTomato,-EGFP)Luo/J reporter mice (Rosa26-mTmG; JAX Stock# 007676) to generate Myh11-CreERT2;Rosa26-mTmG dual reporter mice. To induce CreER^T2^-mediated recombination in MYH11-expressing smooth muscle cells, tamoxifen was administered at 75 mg per kg of animal mass at a concentration of 20 mg/mL for 5 consecutive days via intraperitoneal injection in filtered corn oil (Millipore Sigma). Following induction, Cre-mediated recombination switches reporter expression from membrane tdTomato to membrane GFP in recombined cells. Mice were harvested within 5 days after the final tamoxifen dose.

### TEVG fabrication and preparation

In the presented method, we use a trilayered electrospun graft composed of elastomers that have been previously published including polyester urethane urea (PEUU) and its 50% sulfobetaine carrying variation, PESBUU-50^32,33,38,39^. In both cases, these polymers were prepared in-house as described in Ye, et al. from 2014^32^. This trilayered design consists of an inner PESBUU-50 layer, a middle PEUU:Gelatin (80:20) layer, and an outer PEUU:Gelatin (20:80) layer. This specific TEVG design was chosen to parallel our ongoing rat aortic interpositional implantation studies, and PESBUU-50’s specific role was to provide an antithrombogenic elastomeric inner layer. The TEVGs were electrospun using a temperature, voltage, and humidity-controlled IME electrospinning system (Vivolta, Netherlands). The settings to fabricate the TEVGs are included in Extended Data Table 2. The trilayered TEVGs were crosslinked in 0.5% genipin in 200 proof EtOH for 24 h at 37°C and washed 3x in 200 proof EtOH. Prior to use, TEVGs were sterilized with 70% EtOH, rinsed 3x in sterile 1x DPBS, incubated in sterile 1x DPBS for 12 h. Samples were then rinsed in sterile 1x DPBS immediately before use.

### *Ex vivo* circumferential mechanical compliance testing

Mechanical compliance testing was performed on a closed-end tubular biaxial mechanical loading device (CellScale Biomaterials Testing, Waterloo, Canada). Characterization of rat abdominal aortas was performed *ex vivo* with testing parameters matched between explanted aortas and electrospun scaffolds. Prior to testing, aortas were explanted, cleared of peri-adipose tissue, and flushed clear of blood clots. Aortas and TEVGs were washed with 1xDPBS heated to 37°C and allowed to acclimate for 20 mins prior to testing. Prior to testing, the tubular mechanical testing device was set-up by focusing the camera to accentuate the outer diameter of the samples and assigning size/scale calibration. The sample bath was filled & maintained with 37°C 1x DPBS (aortas in 1x Krebs-Henseleit passive buffer to deactivate vSMCS) and acclimated for 30 mins. The system was then loaded with empty sample holders & load/pressure were tared. The aorta or TEVG was cannulated to sample holders using suture thread upon the ends of the holders and fully sealed from leakage using UV curable glue. Each sample was then loaded & tested using the parameters in Extended Table 3. Preconditioning was set for 9 cycles, and the 10th cycle was recorded as the test.

Compliance Calculations: Data was collected across taut axial stretch to match the axial loads in surgical implantation (generally, λ = 1.3–1.5 for aortas, and further defined by an axial load of 0.10 ±0.02 N. Compliance for our studies was defined as the mean ± 1σ of tested rat aortas at identical conditions. Compliance is calculated for each λ per bio-replicate, computed over ranges of 0.1 mmHg through 120 mmHg by the equation displayed at the bottom of Extended Data Table 3. [(OD120-OD70)/OD70]/OD70, which yields the ratio of the outer diameter (OD) differences when the lumen is pressurized at 70- vs. 120 mmHg. Results are reported as means ± 1σ. Tests with temp. drift >2°C, pressure >2 mmHg & slip/buckling were excluded.

### 3D artery-TEVG culture assembly and maintenance

The complete 3D culture constructs were assembled by combining segments of aorta (each 5 mm length; OD ~1.2 mm, and wall thickness ~0.10 mm) positioned adjacent to a 5 mm-long TEVG with OD/ID closely matched to the aortic segments, both upon a 500 μm polytetrafluoroethylene (PTFE)-coated mandrel. The wire mandrel with construct is then suspended on height-matched 3D printed PC holders placed at each end of the mandrel to maintain tissue alignment and consistent tissue-TEVG contact. Holder spacing could be adjusted to maintain contact if mild axial tissue contraction occurred during early culture. 100 mm and 60 mm dishes were used for isolation/cleaning steps, following transfer to 6-well plates in 4 mL of complete media for culture. Samples were maintained in standard culture conditions (37 °C, 5% CO_2_).

The lowest point of the assembled culture sample was no more than ~3 mm below the air-media interface. Media was changed every 3 days, and samples were visually inspected daily during the first week to ensure tissue-TEVG contact. If contraction reduced contact between the aorta and TEVG within the first week, the holders were slid inward using sterile tweezers. Unless otherwise indicated, constructs were not joined using sutures, adhesives, or clamps to isolate remodeling outcomes attributable to tissue-TEVG apposition as these connection strategies may be introduced as independent experimental variables.

### Live imaging handling and aseptic practices

For live imaging, samples were handled in a biosafety cabinet to remove them from culture media, briefly washed in pre-heated 37 °C sterile 1x DPBS, and transferred to a 35 mm dish of 37 °C FluoroBrite DMEM supplemented with 1% Pen/Strep and 1% gentamicin. For optimal sample imaging conditions, we used an on-stage incubator system (Oko-Labs, Netherlands). We also verified that a culture incubator is not required and that reagents, such as Live Cell Imaging Solution (Thermo Fisher, USA) can be used for stable room-temperature imaging without CO_2_. Post-imaging, samples were immediately covered and returned to a biosafety cabinet, rinsed in pre-heated 37 °C sterile 1x DPBS, and placed back into standard culture conditions. Samples were imaged at 1-week intervals for ≤ 45 mins per sample. Across studies, we found that the most common risk of culture contamination was from the water-dip objective lens. This can be deterred by vigilant disinfection of the objective lens and the surrounding stage with the appropriate cleaners before and after every use.

### Multiphoton microscopy system hardware and device preparation

Multiphoton microscopy was performed using a TriM Scope II (Miltenyi BioTec, Germany) coupled to an Olympus BX51 upright microscope and a Ti:sapphire femtosecond InSight DeepSee+ Dual laser (Newport, USA). Acquisition and hardware control were performed using manufacturer provided software (ImSpector Pro, v7.5.0). Emission was collected with non-descanned PMTs in a backward (epi) configuration, and images were saved as OME.TIF files. We provide Extended Data Figure 4 for more details on system configuration. Prior to imaging, the system was allowed to equilibrate for at least 20 mins. Desired laser power output was then measured for 5 mins using identical settings and plane as the samples. To minimize the risk of contamination from imaging sessions the microscope stage surfaces and the media-contacting portions of the objective were cleaned at least three times with 70% EtOH. The objective lens was cleaned with 200-proof biological grade EtOH and wiped with lens cleaning tissue (Whatman, Grade 105).

### Second harmonic generation (SHG) and two-photon excited fluorescence (2PEF) imaging

For histological slides, adventitial collagen in rat aortic sections was confirmed to generate SHG using 770–810 nm excitation with 377–395 nm emission detection, consistent with prior reports^40–42^. For live 3D cultures, SHG was acquired at 792 nm excitation and collected using a 395/25 nm emission filter. Using the same excitation wavelength, endogenous 2PEF was collected in parallel through 460/40 nm and 620/60 nm channels; an optional 525/50 nm channel yielded qualitatively similar cell-associated signal to 460/40. In merged images, TEVG autofluorescence was identified by signal in both 460/525 and 620 channels, whereas cells were predominantly enriched in the 460/525 channel^28,29,40^. In this work, 2PEF intensity was used as a cell-associated autofluorescence signal to support visualization/segmentation and was not interpreted as a definitive metabolic readout.

For SHG thresholding, native aortic tissue served as a positive control and TEVG-only cultures served as negative controls to define signal-to-noise-based thresholds. Live imaging settings were selected based on prior reports and practical experience for low-photodamage longitudinal imaging (≤15 mW at the sample, ≤5 μs pixel dwell time, and ≤30 s acquisition per z-plane)^25–29,43–45^. We provide Extended Data Table 5 for our live imaging settings.

### Widefield fluorescence and birefringence imaging

Inverted fluorescence imaging was performed on an EVOS M7000 (Thermo Fisher Scientific, USA) using the DAPI v2, GFP v2, and RFP v2 filter cubes. Upright fluorescence and picrosirius red birefringence imaging were performed on a Nikon Eclipse 90i using an in-house linear polarizer. Further details and imaging settings are provided in Extended Data Table 6.

### RNA isolation and qRT-PCR

For gene expression assays, the TEVG portion of the constructs (excluding the original aortic tissue) were rinsed in sterile 1x DPBS and stored in RNAlater (Ambion) at 4°C for ≥24 h, transferred to TRIzol (Invitrogen), and stored at −80°C. Samples were homogenized on ice using Biomasher II tubes with a motorized pestle. Total RNA was isolated by TRIzol/chloroform extraction, and the aqueous phase was purified using the RNeasy Micro Kit PLUS (Qiagen). RNA concentrations were measured with a NanoDrop One (Thermo Fisher Scientific). RNA inputs were normalized and reverse transcribed using SuperScript IV VILO Master Mix (Invitrogen). Quantitative RT-PCR was performed on a QuantStudio 3 (Applied Biosystems, USA) using TaqMan single-plex MGB-FAM and TaqMan Gene Expression Master Mix in 20 μL reactions (MicroAmp Optical 96-well plates) using manufacturer-recommended cycling conditions. Relative-fold expression was calculated using the Livak ΔΔCt method (2^−ΔΔCt^), normalized to controls using the most consistent endogenous reference gene found (*Rer1*), and reported as relative quantification (RQ)^46^.

### SHG fiber quantification and image processing

All initial image processing was performed using ImageJ (FIJI) using version 1.54r, with some downstream assessments in MATLAB. As image post-processing techniques can vary across investigators, we utilized a previously published SHG fiber analysis application known as CT-FIRE (v3.0) to assess maximum intensity projections (MIP) taken from the (Ex: 792 nm) 395/25 channel z-stacks. CT-FIRE was used to extract SHG-positive fibers and quantify fiber width, length, orientation (0–180°), and straightness. CT-FIRE outputs were processed with custom MATLAB scripts to remove invalid entries, convert values to physical units using the imaging pixel size, apply consistent binning/ranges across groups, and compute per-sample summary values with probability-normalized histograms (bin counts divided by the total number of fibers per sample) to enable distribution-shape comparison across groups. All other image post-processing was performed using ImageJ/FIJI. CT-FIRE-based collagen fiber quantification (SHG). For all CT-FIRE analyses, we modified the following two settings from default: minimum fiber length: 15 pixels and maximum fiber width at 30 pixels.

In Figure 5, each sample (biological replicate) was individually analyzed, and CT-FIRE outputs were exported as histograms/arrays describing fiber length, width, angle, and straightness. For downstream analysis, CT-FIRE histogram CSV files (HistLEN, HistWID, HistANG, HistSTR) were batch-processed using a custom MATLAB script to harmonize binning across replicates and to generate replicate-level summary metrics. CT-FIRE outputs (pixel units) were converted to microns using the imaging calibration factor (μm/pixel). All statistical analyses were performed on replicate-level summaries (one value per sample) rather than pooled fibers. For each replicate, the median fiber length and median fiber width were calculated across all fibers detected within that sample. Fiber straightness was summarized as the replicate median of the straightness distribution (end-to-end distance/contour length and bounded between 0 and 1). Fiber alignment was quantified as a nematic order parameter (S), computed from the fiber angle distribution as (θ in radians; nematic periodicity 0–180°), yielding values from 0 (isotropic) to 1 (perfect alignment). The number of fibers contributing to each replicate metric was recorded for quality control. Angle distribution visualization (polar plots). To visualize orientation distributions across groups, fiber angles were binned in 10° increments and normalized per replicate to a probability distribution (each replicate contributed equally). Replicate distributions were averaged within each experimental group to obtain a group mean distribution. As collagen fiber orientation is nematic (0–180°), the one-sided distributions were mirrored to 0–360° for polar visualization graphs.

Prior reports have noted that collagen-based SHG can be polarization and orientation dependent, such that rotating a sample with anisotropic fibers by 90° relative to a fixed excitation polarization can alter SHG intensity and shift the apparent fiber-angle distribution by ~90° ^35,36^. We verified subtle differences in fiber quantification and SHG intensity when samples were imaged at a ~90° orientation with the same acquisition settings (Extended Data Fig. 8). While these were relatively small changes with the samples remaining globally comparable, it motivated us to assign a single sample orientation for all images. We standardized our sample imaging orientation to be at 0° (placed with the length of the sample in a left-to-right orientation with respect to the microscope), and we recommend users of this methodology to standardize identifying their system’s polarization axis and assign a single specimen orientation across all imaging sessions included in a single study.

### Transforming Growth Factor Beta (TGF-β) isoform studies

For the TGF-β studies, carrier-free human recombinant (rh)TGF-β isoforms were reconstituted following the manufacturer’s recommendations at 20 μg/mL in sterile 4 mM HCl, aliquoted, stored in −20°C, with minimal freeze-thaw cycles per aliquot. All isoforms were purchased with matched vendor reported bioactivity levels. Treatments were provided at a concentration of 10 ng/mL of TGF-β into 4mL of media and vehicle controls were given an equal volume (2 μL) in the equivalent 4 mL of media.

### Preparation of alternative TEVGs used in the 3D culture

Polycaprolactone (PCL), PEUU, PESBUU-50, and gelatin TEVGs were electrospun in-house using the previously mentioned system. Electrospinning settings are provided in Extended Data Table 7. To deter degradation, gelatin TEVGs were crosslinked using 0.5% genipin for 24 h at 37°C. For urinary bladder matrix (UBM) hydrogel grafts, an inner 100% PCL sheet was first prepared by spin-casting a 10% w/v PCL (80,000 Mn) in HFIP solution onto glass to a thickness of ~25 μm. UBM was prepared by the Hussey laboratory as previously described^47^. Porcine urinary bladders from market-weight animals were acquired from Tissue Source, LLC. Briefly, the tunica serosa, tunica muscularis externa, tunica submucosa, and tunica muscularis mucosa were mechanically removed. The luminal urothelial cells of the tunica mucosa were dissociated from the basement membrane by washing with deionized water. The remaining tissue consisted of basement membrane and subjacent lamina propria of the tunica mucosa and was decellularized by agitation in 0.1% peracetic acid with 4% EtOH for 2 h at 300 rpm on an orbital shaker. The tissue was then extensively rinsed with PBS and sterile water. The UBM was then lyophilized and milled into particulate using a Wiley Mill with a #60 mesh screen. To prepare UBM-hydrogels, we followed previously published methods^48^. Briefly, decellularized porcine UBM powder was solubilized at a concentration of 20 mg/mL in a solution of 1.0 mg/mL pepsin in 0.01 N HCl, with constant stirring for 24 h. The digest was then neutralized with 0.1 N NaOH, 1x PBS, and 10x PBS to a pH of ~7.4, and a final concentration of 10 mg/mL. The samples were incubated at 37°C for 1 h for gelation, and then immediately applied to the PCL sheets as a thin uniform layer using a cell scraper in a biosafety cabinet. The UBM hydrogel-coated sheet was then manually rolled three times around a 1.1 mm diameter mandrel with coated surface facing outward. The seam was quickly sealed by pressing one side onto a 60°C hot plate for <1 s.

### Statistical and Reproducibility

Statistical analyses and plots were performed in MATLAB. For gene expression results: For each gene, Log_2_(RQ) values were compared among groups using ordinary one-way ANOVA. Normality was assessed on the Log_2_ scale by Shapiro-Wilk test and homogeneity of variance by Brown-Forsythe test. Multiple comparisons used Tukey’s post-hoc test (Tukey-Kramer adjustment for unequal n). Two-sided α = 0.05. Data are presented as mean ± SD with individual biological replicates shown. Technical replicates were averaged prior to analysis.

For CT-FIRE fiber analyses, replicate medians (one point per biological replicate) were plotted by group in GraphPad Prism. Group summaries were displayed as mean ± SD (as specified) across replicate medians, with individual replicate points overlaid. Group differences were tested using one-way ANOVA with Tukey’s multiple comparisons, with statistical significance defined as p < 0.05.

## Supplementary Material

Supplement 1
