# Supplementary material for: Organotypic artery-graft culture enables label-free multiphoton tracking of remodeling that links to long-term graft microarchitecture
Source: bioRxiv. 2026 Mar 3:2026.02.28.708759. Preprint. [Version 1] doi: 10.64898/2026.02.28.708759 (PMC13001454; doi:10.64898/2026.02.28.708759)
Supplement: Supplementary file 1 [file media-1.pdf]

Organotypic artery-graft culture enables label-free multiphoton tracking of remodeling that links to long-term graft microarchitecture

David R. Maestas, Jr., PhD[1], Trin Murphy[1], Katarina M. Martinet, BS[1], Tracey Moyston, BS[1], Leon Xuanyu Min, BS[1,2], Ali Behrangzade, PhD[1], Brock J. Pemberton, BS[1], Sang-Ho Ye, PhD[1], George Hussey, PhD[3,4], Mohamad Azhar, PhD[5,6], William R. Wagner, PhD[1,7,8], Jonathan P. Vande Geest, PhD[1,9]✉

[1]Department of Bioengineering, University of Pittsburgh, Pittsburgh, PA, USA
[2]Department of Medicine, University of Pittsburgh, Pittsburgh, PA, USA
[3]Department of Surgery, University of Pittsburgh, Pittsburgh, PA, USA
[4]McGowan Institute of Regenerative Medicine, University of Pittsburgh, Pittsburgh, PA, USA
[5]Department of Cell Biology & Anatomy, School of Medicine, University of South Carolina, Columbia, South Carolina, USA
[6]Department of Biomedical Engineering Program, University of South Carolina, Columbia, South Carolina, USA
[7]Department of Chemical and Petroleum Engineering, University of Pittsburgh, Pittsburgh, PA, USA
[8]Department of Mechanical Engineering and Materials Science, University of Pittsburgh, Pittsburgh, PA, USA
[9]Vascular Medicine Institute, University of Pittsburgh, Pittsburgh, PA, USA
✉ Email: jpv20@pitt.edu

Extended Data

1. **Extended Data Figures**
Extended Data Figure 1: Label-Free Multiphoton Imaging of Aortic Sections & Cultures
Extended Data Figure 2: Label-free Multiphoton Imaging of an *Ex Vivo* Aortic Injury Model
Extended Data Figure 3: 3D Culture Aorta-Graft Dimensional Matching
Extended Data Figure 4: Multiphoton Optical Configuration
Extended Data Figure 5: 3D Culture Platform Compatibility with Common TEVG Biomaterials
Extended Data Figure 6: Tracking SHG with a 4x Objective Lens
Extended Data Figure 7: Appearance of Cultures Under Standard Microscopes & 2PEF Validation
Extended Data Figure 8: Sample Orientation Impacts SHG Outcomes
Extended Data Figure 9: Murine & Reporter-Strain Compatibility in the 3D Culture Platform
Extended Data Figure 10: TGF-β Isoform Extended Cultures

2. **Extended Data Tables**
Extended Data Table 1: Reagents and supplies
Extended Data Table 2: Electrospinning parameters for trilayered TEVGs
Extended Data Table 3: Tubular mechanical compliance testing
Extended Data Table 4: Optical filters, PMTs, and imaging settings
Extended Data Table 5: Live imaging settings (16x Objective)
Extended Data Table 6: EVOS M7000 inverted widefield microscope configuration
Extended Data Table 7: Alternative biomaterial TEVG fabrication settings

3. **.STL File for 3D Printable Hexagon Sample Holders**

Extended Data Figure 1

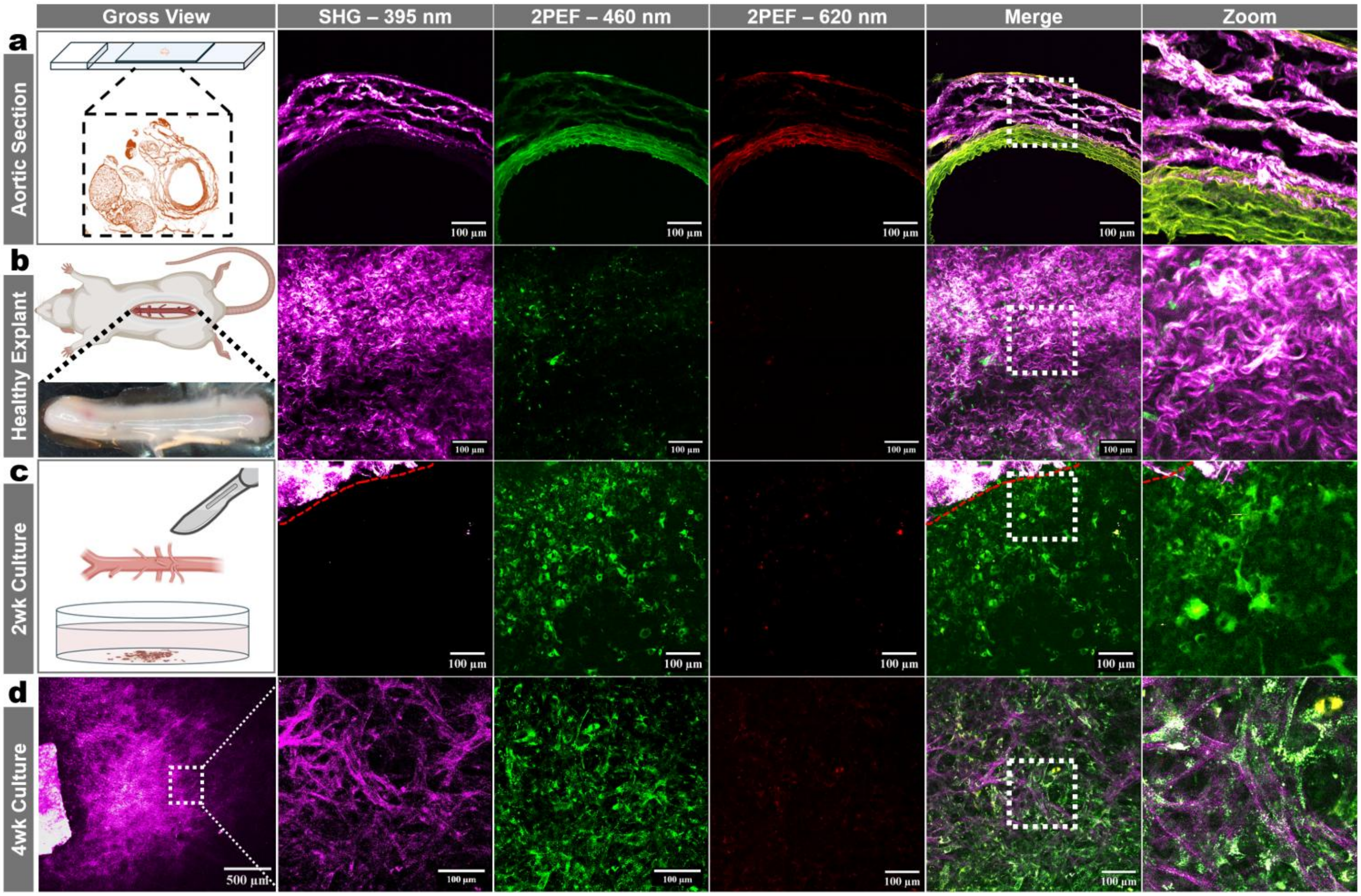

Extended Data Fig. 1 | Label-Free Multiphoton Imaging of Aortic Sections & Cultures. a. An abdominal aortic explant section from a male rat imaged under a multiphoton microscope. **b.** Live multiphoton 3D stack images of an explanted rat aorta. **c.** A live multiphoton image of an early rat aortic explant that was diced and cultured in smooth muscle cell media (SmGM-2) for 2-weeks. Top left of image shows a piece of the cut portion of aorta in view. **d.** A live multiphoton image taken from a longer-term aortic explant culture (4wks) showing high levels of cellular-associated 2PEF and collagen-associated SHG. For each example shown, samples were excited at ~800 nm laser wavelength with collection across three channels simultaneously: 395/25 nm (SHG), 460/40 nm (cell-associated 2PEF), and 620/60 nm (broad 2PEF). Scale bars are 100 µm.

Extended Data Figure 2:

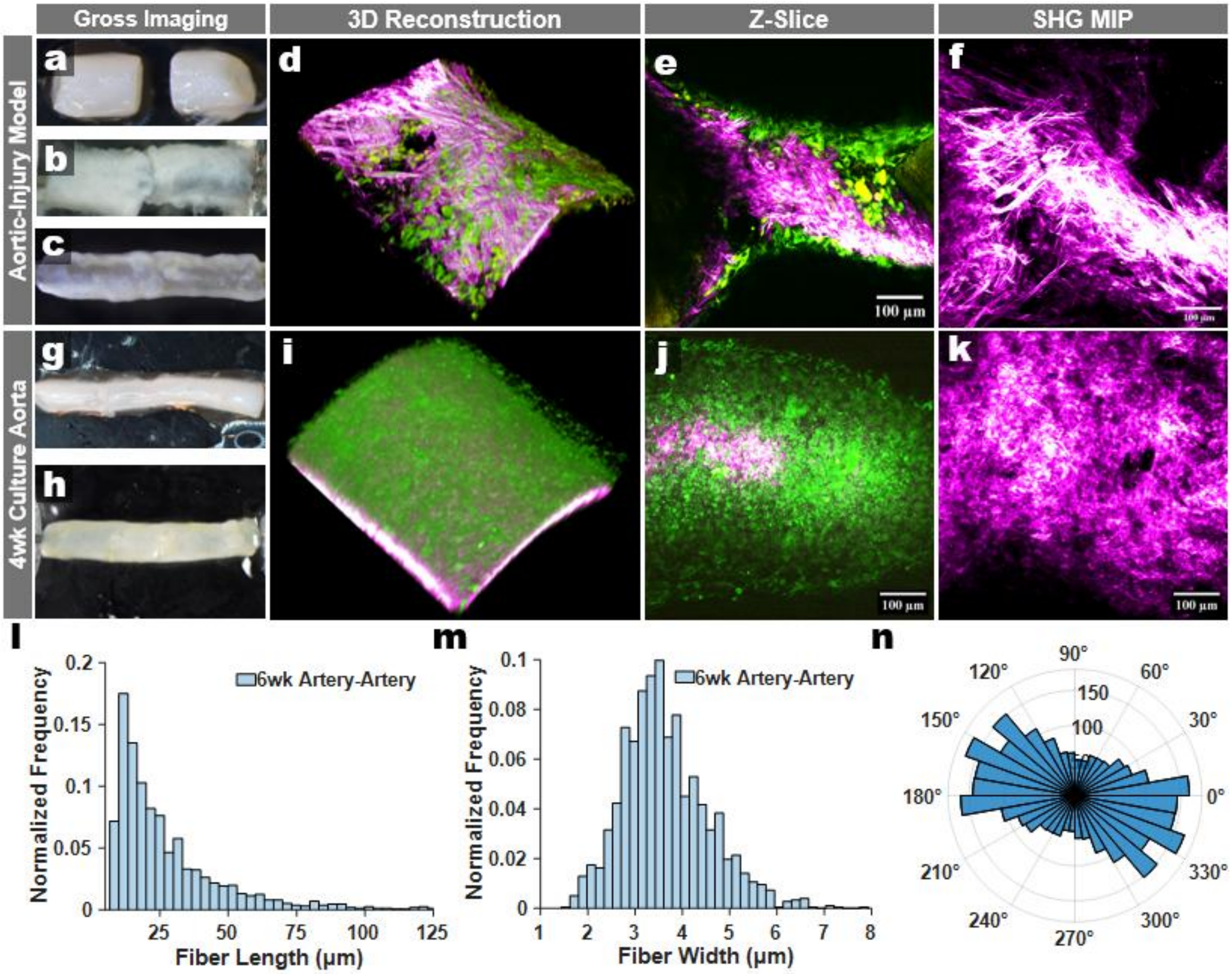

Extended Data Fig. 2 | Label-free Multiphoton Imaging of an *Ex Vivo* Aortic Injury Model. a-c. Gross images of the aortic injury model construct during preparation and after culture (artery–artery repair configuration). **d.** 3D volume rendering of the injury-site region showing SHG collagen signal (magenta hot) overlaid with cell-associated 2PEF as green. **e.** Representative single optical z-slice through the injury region. **f.** SHG maximum-intensity projection (MIP) highlighting fibrillar collagen organization at the injury interface. **g-h**. Gross images of a healthy (uninjured) aorta maintained in 4-week static culture as a reference tissue. **i.** 3D volume rendering of the cultured healthy aorta (SHG, magenta hot and 2PEF as green). **j**. Representative z-slice. **k.** SHG MIP of fibrillar collagen structure in the 4-week cultured healthy aorta. **l-n**. SHG fiber metrics quantified from the aortic injury model (6-week artery–artery condition) using established SHG fiber-extraction analysis (e.g., CT-FIRE): (**l**) fiber length distribution, (**m**) fiber width distribution, and (**n**) fiber orientation distribution (polar histogram). Scale bars, 100 µm (**e, f, j, k**).

Extended Data Figure 3:

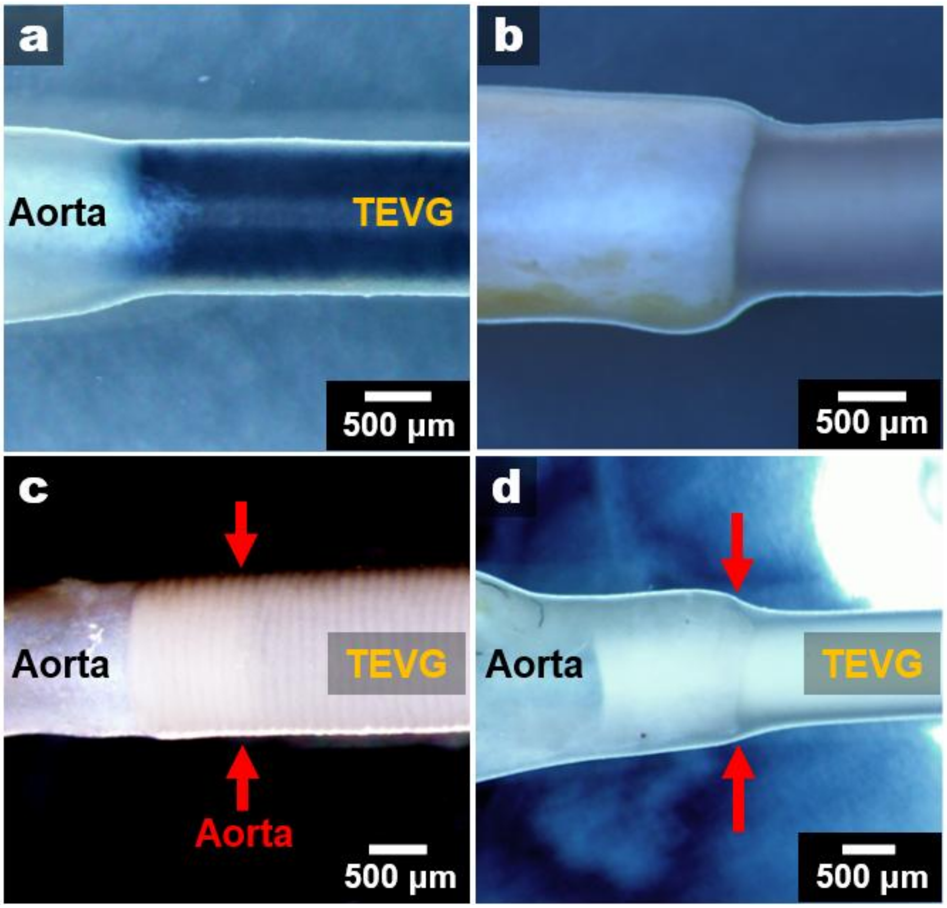

Extended Data Fig. 3 | 3D Culture Aorta-Graft Dimensional Matching. Gross images from various 3D cultures with different aorta-TEVG sizes. **a.** Demonstration of a dimensionally well-matched TEVG and aorta in culture, allowing for monitoring of the graft without ingrowth/outgrowth confounders. **b.** An example of a TEVG that is mismatched, but within tolerance, allowing for multiphoton imaging of cellularization and remodeling on the graft's outer surface. **c**. Depiction of a TEVG used in the culture with a significantly larger outer diameter, causing the aorta to grow on the graft's exterior. **d.** A vascular graft that is too small in outer diameter compared to the aorta. All grafts depicted are at 4-8 weeks in culture. Red arrows are pointing to the areas of ingrowth/outgrowth when mismatched in size by more than 30%.

Extended Data Figure 4:

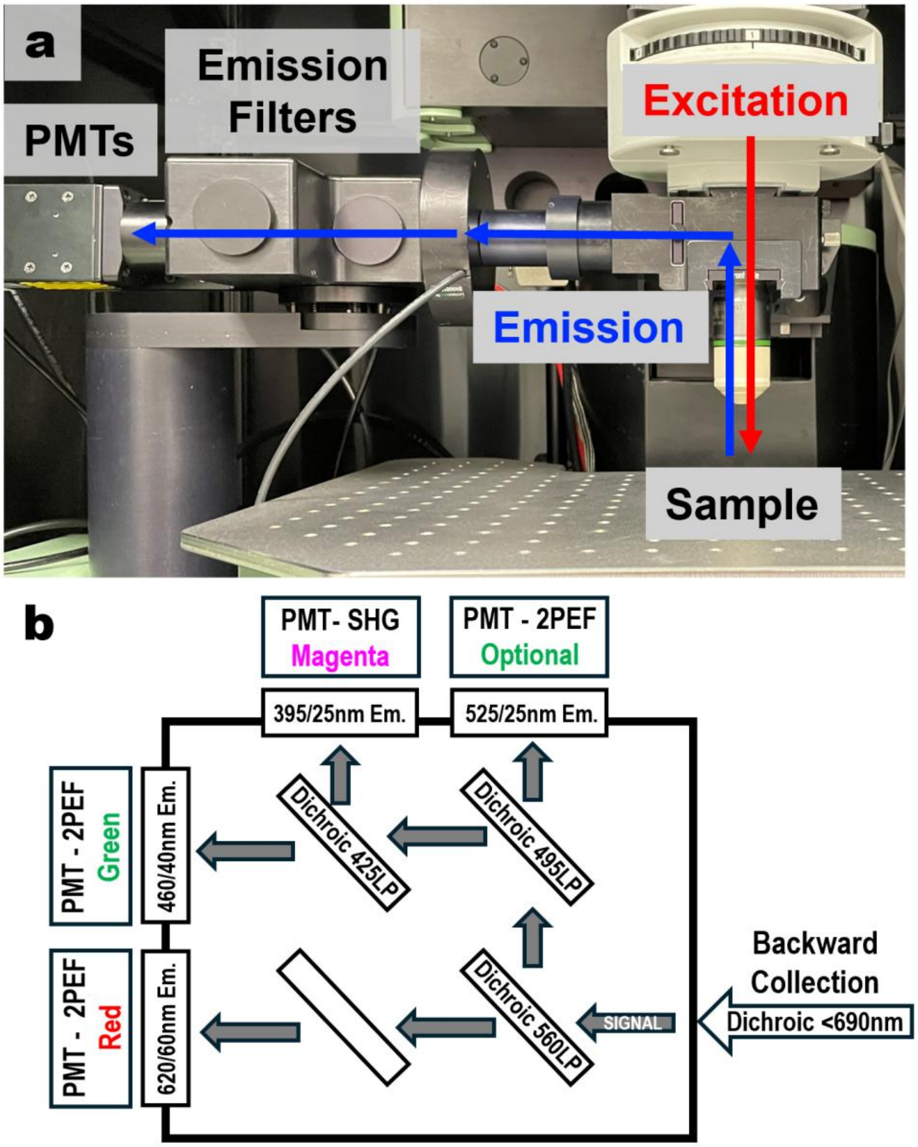

Extended Data Fig. 4 | Multiphoton Optical Configuration. a. Depiction of the gross imaging set up and light paths. **b.** A schematic of dichroic filters used for signal acquisition. The system is designed for epi (backwards) collection of SHG and 2PEF due to 3D sample thicknesses.

Extended Data Figure 5:

The platform supports multiple scaffold biomaterial choices

To assess broader applicability beyond our trilayered TEVGs, we utilized our platform to investigate electrospun grafts made from biomaterials commonly used in vascular research. As before, we assembled culture constructs and combined them with alternative biomaterials (gelatin, UBM hydrogel, PEUU, and PESBUU-50) and imaged them under matched conditions and acquisition settings for 4 weeks in reference to our original trilayered designs (**Extended Fig. 5a**). We found that electrospun porcine-derived gelatin grafts (crosslinked with genipin) exhibited high levels of cellular 2PEF and little to no SHG by 4 weeks. Notably, gelatin grafts required a 4th collection channel (525/50 nm) to distinguish the graft from the cellular 2PEF signatures (**Extended Fig. 5b**). We found that electrospun PESBUU-50, a zwitterionic sulfobetaine-modified PEUU, exhibited high levels of cellular 2PEF and little to no SHG signatures by 4 weeks (**Extended Fig. 5d**). We found that other natural biomaterials, such as decellularized porcine urinary bladder matrix (UBM), may obscure tracking of *de novo* collagen remodeling. Using established protocols, we prepared and coated UBM hydrogels upon the outer surface of a rolled spin-casted sheet of PCL and cultured the constructs as before for 4 weeks. In agreement with prior reports, the UBM-hydrogels retained SHG signal in disorganized bundle-like shapes, suggesting that the use of ECM products for SHG tracking in our platform may require alternative preparation protocols[1]. While we could not confidently distinguish de novo SHG signals, we found that the UBM-hydrogel cultures exhibited strong cellular 2PEF. In contrast, we found that electrospun PEUU grafts, which lack sulfobetaine content, exhibited robust increases in SHG and relatively low levels of cellular 2PEF (**Extended Fig. 5e**). We then evaluated electrospun PCL, a polymer known to lack 2PEF signatures at ~800 nm excitation and found that it lacked TEVG-specific 2PEF, had minimal cellular 2PEF and that the SHG signatures could still be monitored along the graft's surfaces. PCL TEVGs had substantially lower levels of cellular 2PEF, but very high SHG levels (**Extended Fig. 5f**).

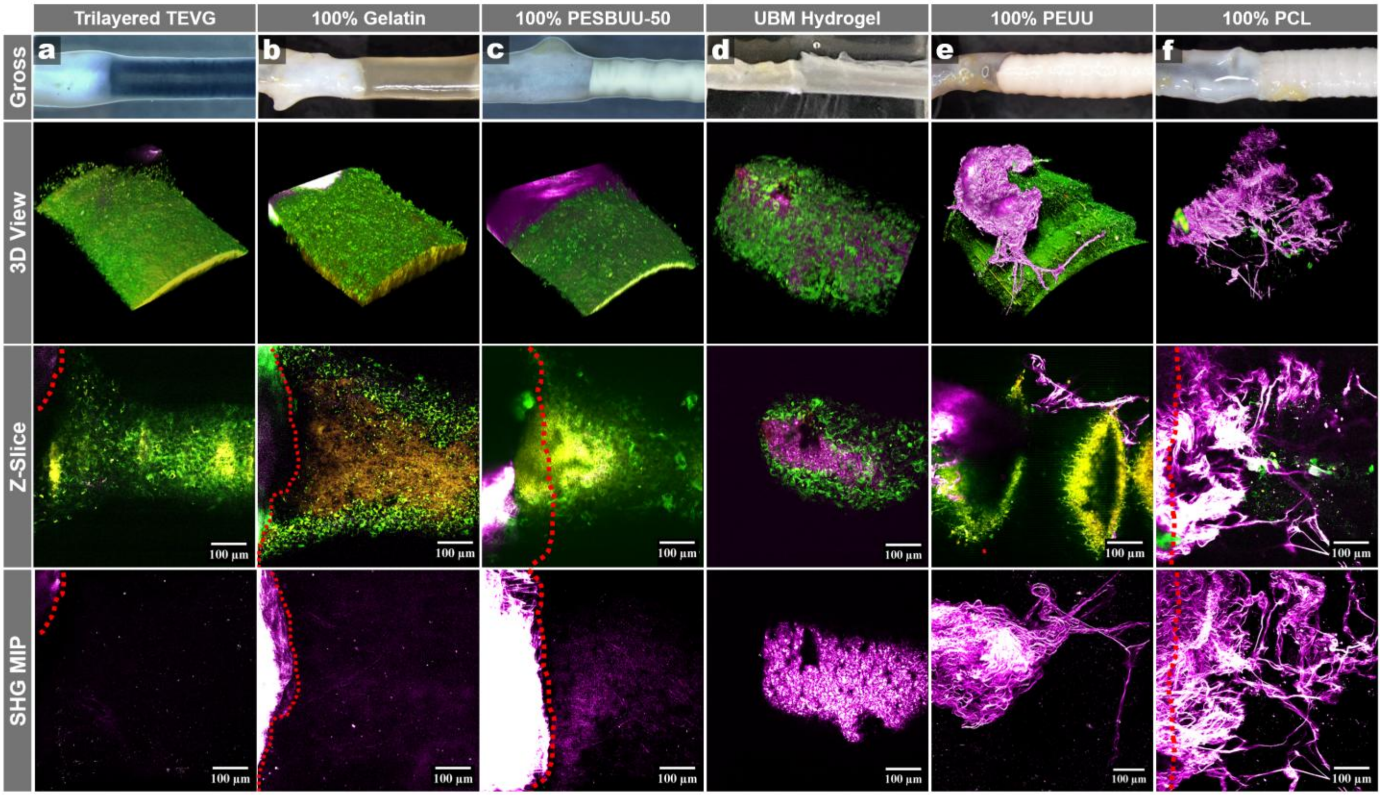

Extended Data Fig. 5 | 3D Culture Platform Compatibility with Common TEVG Biomaterials. a-f. Representative longitudinal live multiphoton imaging of graft interfaces in the 3D *ex vivo* culture at the 4wk timepoint. Images are displayed as merged channels under 792 nm excitation, showing SHG (magenta), cellular 2PEF (green), and TEVG (when applicable, yellow/orange). For each is shown a 3D reconstruction (top), representative z-slice (middle), and SHG MIP (bottom). **a.** Images from the previous 4wk cultured trilayered TEVG, as a reference. **b.** An electrospun gelatin scaffold (crosslinked with 0.5% genipin for 24h in EtOH). 460/40 nm (orange) to identify the graft and overlap between 525/50 nm and 620/60 nm for cellular 2PEF (green). **c.** An electrospun PESBUU-50 TEVG cultured for 4wks. **d.** A spin casted PCL sheet (~25 µm in thickness) coated with a UBM hydrogel and wrapped into a TEVG cylinder at 4wks. **e.** Images from an electrospun PEUU TEVG at 4wks. **f.** An electrospun polycaprolactone (PCL) TEVG. Red dashed lines (bottom row) indicate areas where the aorta is included in the view. Scale bars are 100 µm.

Extended Data Figure 6:

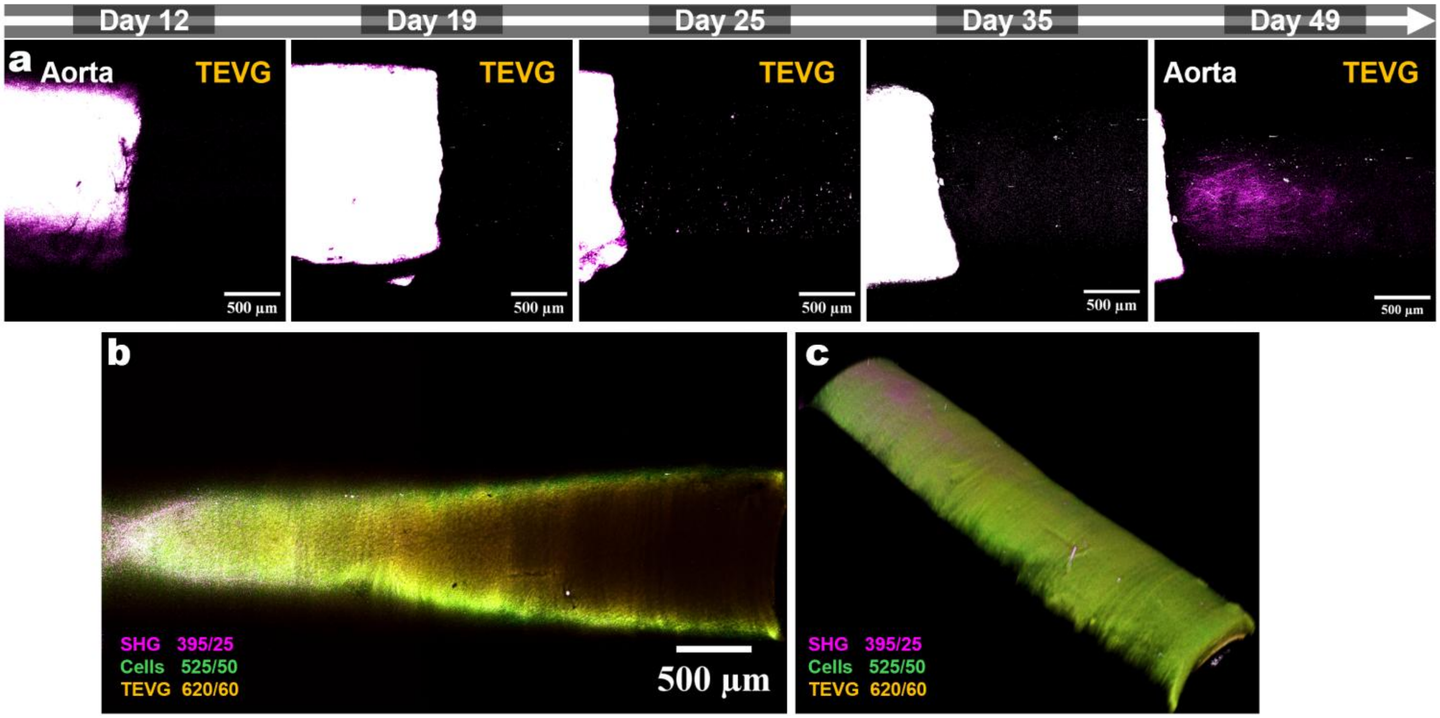

Extended Data Fig. 6 | Tracking SHG with a 4x Objective Lens. a. Representative images SHG (only) across time using a low-NA and low-resolution objective lens (4x). **b**. A representative z-slice image from a z-stack showing all three signals using a 4x objective. **c.** 3D reconstructions of a mosaic of 3D stack images taken of an 8-week cultured sample (single cannulation to 1 aorta) at 4x. Panels (**b**) and (**c**) includes all 3 channels as noted on the images.

Extended Data Figure 7:

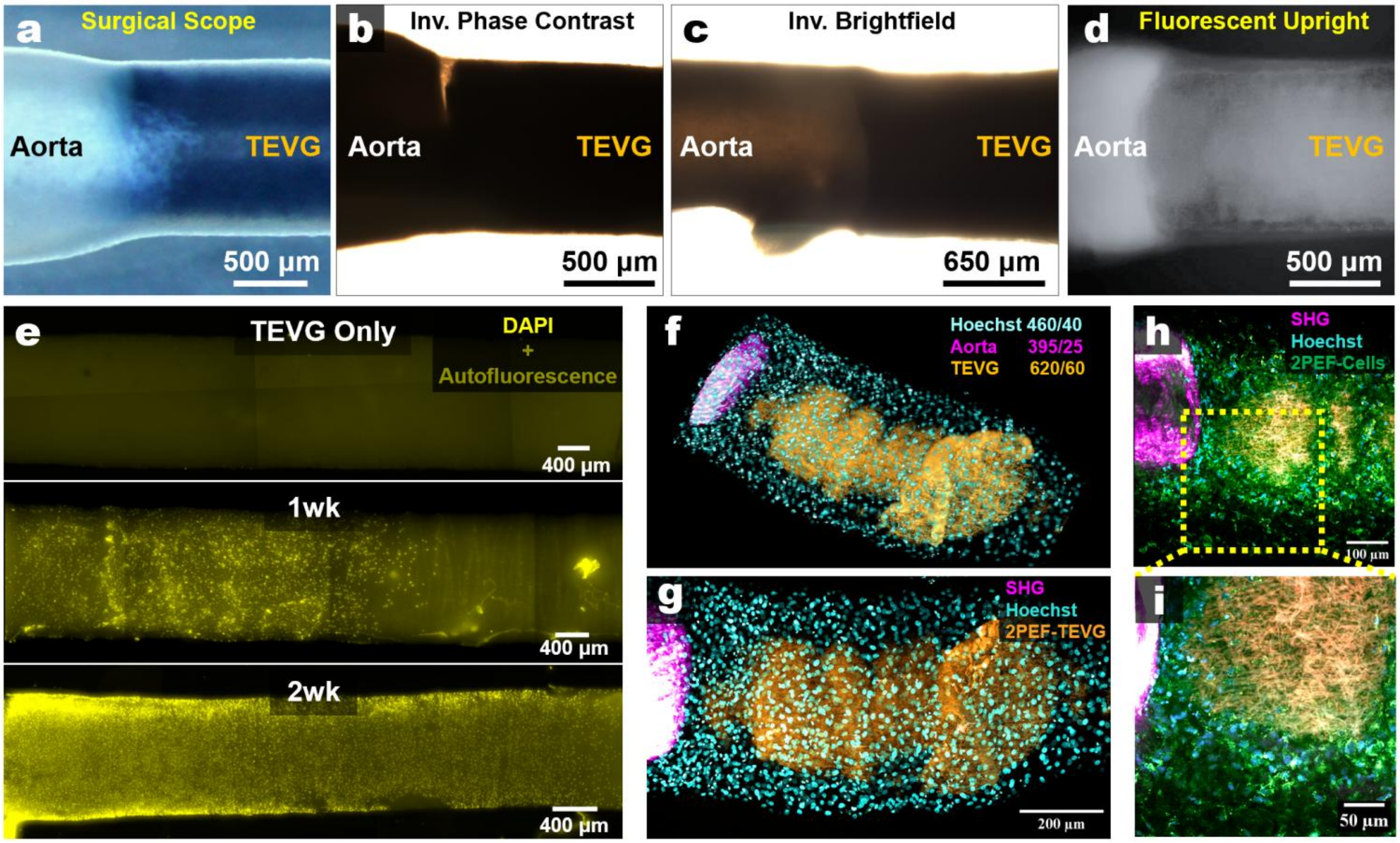

Extended Data Fig. 7 | Appearance of Cultures Under Standard Microscopes & 2PEF Validation. Representative images of how the culture model appears when unstained under various illuminations. **a**. Depicts an 8-week sample under a dissection/surgical microscope at 4x. **b**. Shows a sample under inverted phase contrast lighting. This enables viewing of immature graft interfaces at 4x, slightly zoomed. **c.** Shows a sample under inverted brightfield illumination at 4x. **d.** Shows a representative sample as viewed on an upright scope after halogen lamp illumination and viewed with a dual emission filter for eGFP and mCherry (Chroma filter 59022). **e.** Images of DAPI staining of fixed single-joined (an aorta on one side) trilayered TEVG at 1wk and 2wks in comparison to a TEVG that cultured and DAPI stained without an aorta (top). **f.** A 3D reconstruction of an imaged 2-week cultured sample (double joined with an aorta on each end) stained with Hoechst live cell nuclear dye. **g.** A maximum intensity projection (MIP) of all channels to visualize the cells on the TEVG at 2wks. **h,i.** An image of the same cultured sample now at 8wks post-staining showing 2PEF, Hoechst, and SHG (**h**) with a zoom-in of the region indicated by the yellow dotted lines (**i**).

Extended Data Figure 8:

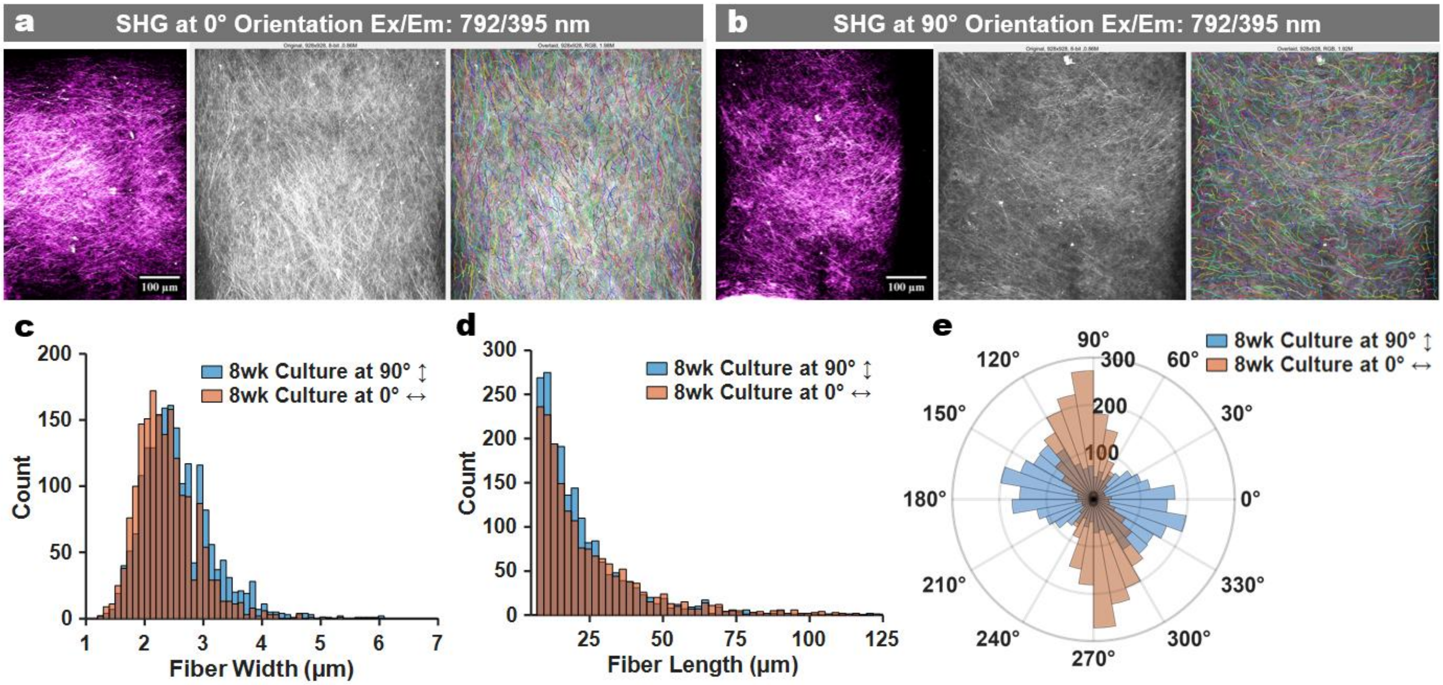

Extended Data Fig. 8 | Sample Orientation Impacts SHG Outcomes. a. Representative SHG channel MIP (Ex/Em:792) (left) with a CT-FIRE overlay of determined fibers (right). **b.** The same sample turned 90 degrees and imaged with the same acquisition settings (noticeably dimmer signal). **c-e.** CT-FIRE analyses of both orientations overlaid for fiber width (**c**), fiber length (**d**), and polar alignment (**e**).

Extended Data Figure 9:

3D culture compatibility with fluorescent reporter strains

To expand the utility of the platform to murine models, we evaluated mouse aortic explants and a dual reporter strain in the 3D culture. We generated a tamoxifen-inducible dual fluorescent reporter to distinguish smooth muscle lineage cells (GFP) from surrounding stromal populations (tdTomato Red) for live imaging by crossing Myh11-CreER^{T2} mice with the mT/mG Cre reporter line. Widefield imaging confirmed reporter signal at both the gross tissue level and in primary murine aortic cells in 2D culture, with minimal background in wild-type controls (**Extended Data Fig. 9a,b**). We found that optical clearing enabled more depth in imaging with widefield fluorescent imaging of explanted aortas, but required fixation, dehydration, and clearing protocols (**Extended Data Fig. 9c, top**). We then used multiphoton imaging to find that the tdTomato and GFP signatures were weak under our standard live imaging acquisition settings, and that the expected 2-photon excitation of 900 nm was sufficient to visualize the GFP and tdTomato red signal within live aortas (**Extended Data Fig. 9c, bottom**). We then verified that the dual reporter aortas could successfully be integrated into the 3D culture model and be imaged using our live acquisition settings to see cells, SHG, and TEVG, as well as utilize different excitations to visualize the GFP$^+$ smooth muscle cells (**Extended Fig 9d**).

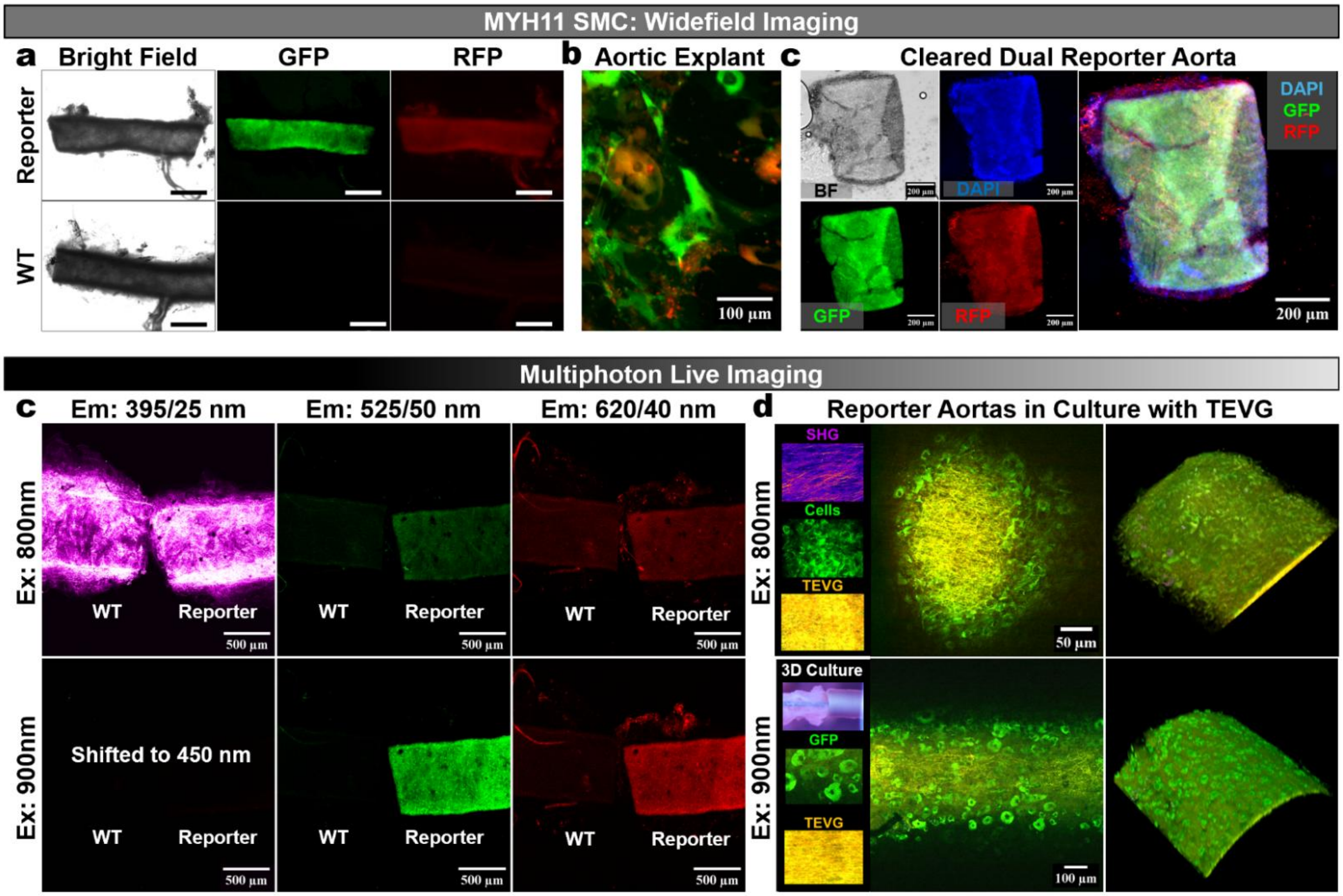

Extended Data Fig. 9 | Murine & Reporter-Strain Compatibility in the 3D Culture Platform. a. Gross validation of a dual-reporter mouse line following tamoxifen induction of Cre recombination, with ubiquitous tdTomato (RFP) labeling of all cells and GFP labeling of MYH11 expressing smooth muscle cells (SMCs). Representative brightfield, GFP, and RFP fluorescence images compare reporter aortic tissue to wild-type (WT) controls to confirm reporter specificity at the tissue scale. **b.** Higher-magnification fluorescence image of primary murine aortic cells in 2D culture demonstrating cellular-level reporter expression and co-localization patterns consistent with the dual-reporter design. **c**. Terminal optical clearing enables limited volumetric imaging of the dual reporter signal under widefield fluorescence, shown as brightfield (BF), DAPI nuclear stain, GFP, RFP, and a merged reconstruction (DAPI/GFP/RFP). **d.** Live multiphoton imaging of WT and reporter aortas to map signatures across the standardized collection channels. Under ~800 nm excitation, signal is shown for SHG (395/25 nm; magenta), the GFP-compatible window (525/50 nm; green), and the tdTomato/RFP-compatible window (620/60 nm; red). **e, top.** Representative z-plane image (left) and 3D reconstruction under ~800 nm excitation showing cellular 2PEF and scaffold 2PEF. **e**, **bottom**. Increasing the excitation wavelength to ~900 nm enhances visualization of the GFP reporter, with corresponding channel readouts shown for comparison. Scale bars in (a) are 1 mm, (c) 200 µm, (d) 500 µm, (e, top) 50 µm, & (e, bottom) 100 µm.

Extended Data Figure 10:

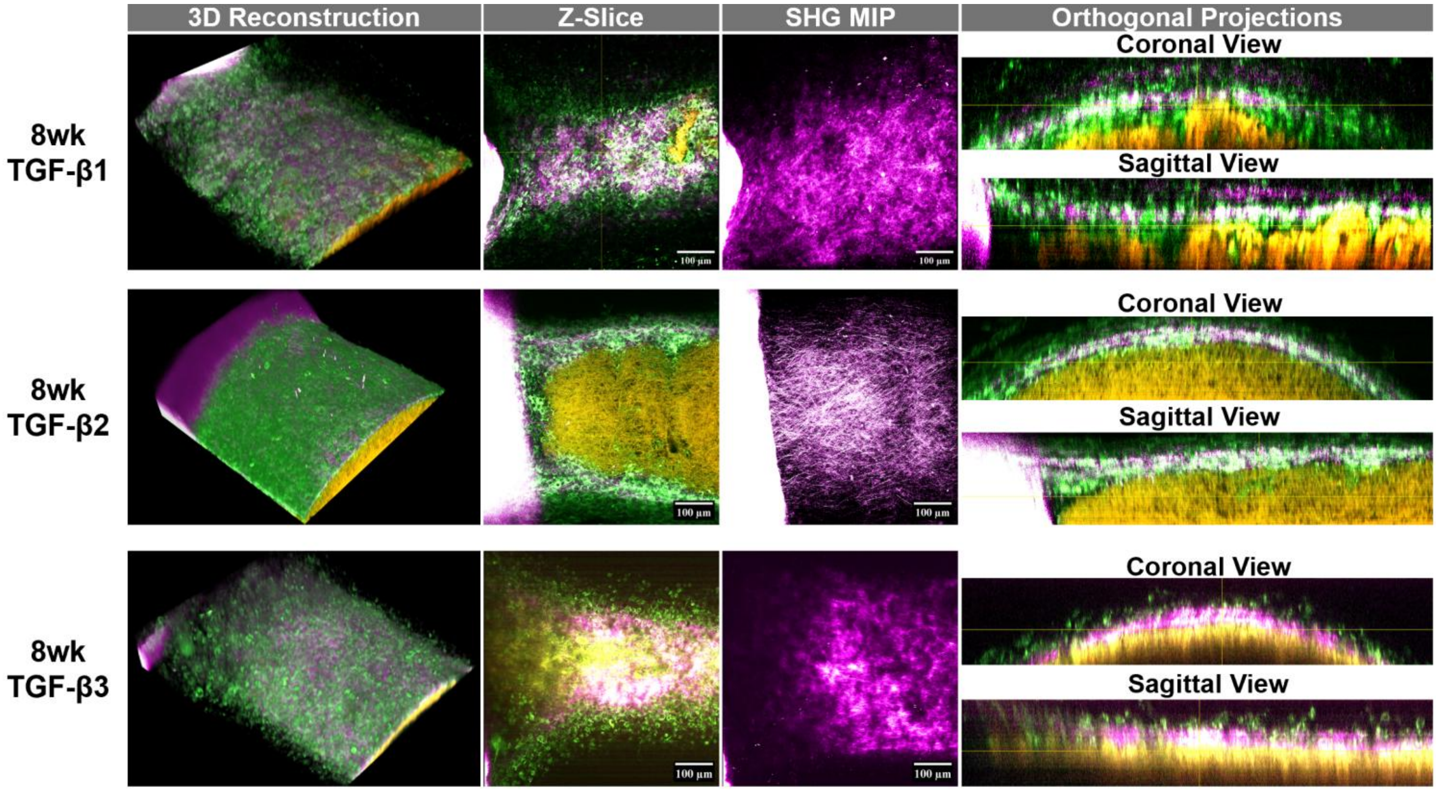

Extended Data Fig. 10 | TGF-β Isoform Extended Cultures. 3D Cultures treated every three days with a TGF-β isoform ceased treatment at 4 weeks and were continued in culture for 4 additional weeks (8 weeks in total). Representative images of each groups general imaging appearances and differences in remodeling signatures.

Extended Data Table 1: Reagents & Supplies

**Vascular Graft Fabrication Supplies**	**Catalog # / ID**	**Vendor**	**Supplier (If Applicable)**
1,1,1,3,3,3-Hexafluoro-2-propanol (HFIP)	H1008	Spectrum Chemical	-
Type A Gelatin from Porcine Skin	G1890	MilliporeSigma	-
Polycaprolactone 80kDa	440744	MilliporeSigma	-
Genipin	6902-77-8	FujiFilm	Fisher Scientific
PEUU	-	-	In-house preparation
PESBUU-50	-	-	In-house preparation
**3D Culture Supplies**	**Catalog # / ID**	**Vendor**	**Supplier (If Applicable)**
SmGm2 Media with Bullet Kit	CC-3182	Lonza	-
PTFE coated mandrels (<0.5mm OD)	206033	Applied Plastics	-
Polycarbonate filament (Ultimaker)	1640	Ultimaker	Dynamism
1x Sterile DPBS	14190235	Gibco	Thermo Fisher Scientific
Pen/Step 10,000 U	15140122	Gibco	Thermo Fisher Scientific
Live Cell Imaging Solution (optional)	A59688DJ	Gibco	Thermo Fisher Scientific
FluoroBrite DMEM	A1896701	Gibco	Thermo Fisher Scientific
Amphotericin B	15290018	Gibco	Thermo Fisher Scientific
100mm x 25mm Petri Dishes	FB0875711	FisherBrand	Fisher Scientific
60mm Petri Dishes	50828744	CELLTREAT	Fisher Scientific
35mm Petri Dishes	627161	Greiner Bio-One	Thermo Fisher Scientific
6-well Plates	140675	Thermo Scientific Nunc	Thermo Fisher Scientific
SylGard 184 Kit (optional PDMS)	4019862	Dow	Krayden
Whatman lens cleaning tissue, Grd. 105	WHA2105841	Whatman	MilliporeSigma
**Proteins for 3D Culture**	**Catalog # / ID**	**Vendor**	**Supplier (If Applicable)**
rhTGFb1 carrier-free	240B010/CF	R&D Systems	-
rhTGFb2 carrier-free	302-B2-010/CF	R&D Systems	-
rhTGFb3 carrier-free	243B3010/CF	R&D Systems	-
**qRT-PCR Gene Expression**	**Catalog # / ID**	**Vendor**	**Supplier (If Applicable)**
RNALater	AM7020	Ambion	Thermo Fisher Scientific
Trizol Reagent	15596018	Invitrogen	Thermo Fisher Scientific
Biomasher 2 (pestles+tubes)	K7496250030	Nippi	Fisher Scientific
RNA suitable Chloroform	C2432-4X25ML	MilliporeSigma	-
RNEasy Microkit PLUS	74034	Qiagen	-
TaqMan Master Mix	4370048	Applied Biosystems	Thermo Fisher Scientific
0.2mL PCR Tube Strips	14230215	FisherBrand	Fisher Scientific
SuperScript IV VILO	11756050	Invitrogen	Thermo Fisher Scientific
DNA LoBind 0.5mL tubes	05-414-202	Eppendorf	Fisher Scientific
Optical Film	4311971	Applied Biosystems	Thermo Fisher Scientific
Optical Plates	N8010560	Applied Biosystems	Thermo Fisher Scientific
TaqMan Probe: *Rer1* (MGB-FAM)	Rn01516516_m1	Applied Biosystems	Thermo Fisher Scientific
TaqMan Probe: Hprt1 (MGB-FAM)	Rn01527840_m1	Applied Biosystems	Thermo Fisher Scientific
TaqMan Probe: Gapdh (MGB-FAM)	Rn01775763_g1	Applied Biosystems	Thermo Fisher Scientific
TaqMan Probe: Acta2 (MGB-FAM)	Rn01759928_g1	Applied Biosystems	Thermo Fisher Scientific
TaqMan Probe: Myh11 (MGB-FAM)	Rn01530321_m1	Applied Biosystems	Thermo Fisher Scientific
TaqMan Probe: Lox (MGB-FAM)	Rn01491829_m1	Applied Biosystems	Thermo Fisher Scientific
TaqMan Probe: Col1a1 (MGB-FAM)	Rn01463848_m1	Applied Biosystems	Thermo Fisher Scientific
TaqMan Probe: Col3a1 (MGB-FAM)	Rn01437681_m1	Applied Biosystems	Thermo Fisher Scientific
**Histological Staining**	**Catalog # / ID**	**Vendor**	**Supplier (If Applicable)**
Rabbit Anti-Rat-Vimentin	ab92547	Abcam	-
Rabbit Anti-Rat-MYHC11	ab224804	Abcam	-
Goat anti-Rabbit IgG (AF488)	ab150077	Abcam	-
Picrosirius Red Stain (Connective Tissue)	ab150681	Abcam	-
**Immunofluorescent Staining/Dyes**	**Catalog # / ID**	**Vendor**	**Supplier (If Applicable)**
NucBlue Live ReadyProbes Hoechst 33342	R37605	Invitrogen	Thermo Fisher Scientific
NucBlue Fixed Cell ReadyProbes DAPI	R37606	Invitrogen	Thermo Fisher Scientific

Extended Data Table 2: Electrospinning Parameters for Trilayered TEVGs

Layer	Lumen	Media	Outer
Polymer	PESBUU-50	PEUU:Gelatin	PEUU:Gelatin
Polymer Ratio	100 %	80:20	20:80
Conc. (w/v %)	8 % in HFIP	10 % in HFIP	10 % in HFIP
Dispensing Capillary ID	0.81 mm (18G) Stainless Steel	0.62 mm (20G) Stainless Steel	0.62 mm (20G) Stainless Steel
Dispense Volume Dispense Rate	30 µL 10 µL/min	65 µL 10 µL/min	125 µL 10 µL/min
Nozzle Translation Distance & Rate	80 mm 30 mm/s	80 mm 30 mm/s	80 mm 30 mm/s
Mandrel OD	1.1 mm	1.1 mm	1.1 mm
Mandrel Length	150 mm	150 mm	150 mm
Mandrel Coating	PTFE	PTFE	PTFE
Mandrel Rotation	1000 RPM	1000 RPM	1000 RPM
Working Distance	12 cm	10 cm	10 cm
Voltage Settings	+20 kV Nozzle -4 kV Mandrel	+20 kV Nozzle -4 kV Mandrel	+20 kV Nozzle -4 kV Mandrel
Temp. & Humidity	21°C, 30 %	21°C, 30 %	21°C, 40 %
Crosslinking	24hrs in 0.5% Genipin in 200 proof EtOH at 37°C		
Portion Used	Central 50 mm (±25 mm from center)		

Extended Data Table 3: Tubular Biaxial Testing	
**Submerged in**	DPBS or Krebs-Henseleit
**Temperature**	37.0 ± 2.0 °C
**Initial Axial Load**	Taut (~0.010 N)
**Initial Luminal Pressure**	0.1 ± 0.05 mmHg
**Loading Scheme**	Cyclic, Sinusiodal
**Loading Rate(s)**	1 mmHg/s; 0.5mm/s
**Cyclic Applied Pressure**	0.1 to 120.0 ± 1 mmHg
**Sampling Rate**	5 Hz
**No. of PreCond. Cycles**	9
**Controller Type**	PID
**No. of Diameter ROIs**	6 pairs
**Axial Length of ROI**	5 mm

$$\textbf{\textit{Compliance}} = \frac{\left(\frac{OD_{120} - OD_{70}}{OD_{70}}\right)}{(120-70)\ \text{mmHg}}$$

Extended Data Table 4: Multiphoton Hardware, Optical Filters, & PMTs			
**Multiphoton Component**	**Type**	**Vendor**	**Catalog #**
TriM Scope II	Upright	LaVision Biotec	-
InSight Deep See + Dual laser		Newport	InSight DS+
**Photomultiplier Tube (PMT)**	**Type**	**Vendor**	**Catalog #**
Non-descanned PMT 1	GaAsP	Hamamatsu	H7422-40-LV 5M
Non-descanned PMT 2	GaAsP	Hamamatsu	H7422-40-LV 5M
Non-descanned PMT 3	GaAsP	Hamamatsu	H7422-40-LV 5M
Non-descanned PMT 4	Bialkali	Hamamatsu	H6780-01-LV 5M
**Objectives**	**NA**	**Vendor**	**Catalog #**
Olympus XL Fluor 2x	0.14	Olympus	AMEP4751
Olympus XL Fluor 4x	0.28	Olympus	AMEP4979
UM Plan Fl N 10x	0.30	Olympus	1-U2M583
Nikon LWD 16x	0.80	Nikon	CFI75
W Plan-Apochromat 20x	1.00	Zeiss	421452-9980-000
**Filter**	**Type**	**Vendor**	**Catalog #**
377/50 nm	Em	Chroma Technology	ET377/50m
395/25 nm	Em	Chroma Technology	ET395/25x
460/40 nm	Em	Chroma Technology	ET460/40m
525/25 nm	Em	Chroma Technology	ET525/50m
620/60 nm	Em	Chroma Technology	ET620/60m
425LP	DLP	Chroma Technology	T425lpxr
495LP	DLP	Chroma Technology	T495lpxr
560LP	DLP	Chroma Technology	T560lpxr
690LP	DLP	Chroma Technology	T690lpxr
DLP/LP = Dichroic long pass, Em = Emission (bandpass), PMT = Photomultiplier tube			

Extended Data Table 5: Live Imaging Settings 16x	
**Excitation**	792 nm
**Laser Power**	13.5mW
**Time per Z-Slice**	22,265 ms
**Pixel Dwell Time**	4.4 μs
**Scan Frequency**	200 Hz
**Line Averages**	4
**Avg. Z-Depth**	200 μm
**Z-Step Size**	1.5 - 2.0 μm

Extended Data Table 6: EVOS M7000 Optical Filters and Objectives

Filter Cubes	Excitation/Emission	Catalog Number
DAPI	Ex: 357/44 Em: 447/60	AMEP4950
GFP	Ex: 482/25 Em: 524/24	AMEP4951
Texas Red	Ex: 585/29 Em: 628/32	AMEP4955
CY5	Ex: 635/18 Em: 692/40	AMEP4956
RFP	Ex: 542/20 Em: 593/40	AMEP4952
CFP	Ex: 445/45 Em: 510/42	AMEP4953
YFP	Ex: 500/24 Em: 542/27	AMEP4954
CY7	Ex: 716/40 Em: 794/32	AMEP4967
**Objective Lens**	**EVOS M7000 Objective Lens Name**	**Catalog Number**
4x	OBJ FL 4X LWDPH 0.13NA/10.58WD	AMEP4980
10x	OBJ FL 10X LWDPH 0.30NA/7.13WD	AMEP4981
20x	OBJ FL 20X LWDPH 0.45NA/6.12WD	AMEP4982
40x	OBJ OLY FL 40X 0.6/2.7-4.4	AMEP4764EO
60x	OBJ OLY FL 60X 0.9NA/0.2WD	AMEP4849

Extended Data Table 7: Electrospinning Parameters for Single Layer TEVGs				
Alternative TEVGs				
**Polymer**	**PCL**	**Gelatin**	**PEUU**	**PESBUU-50**
**Conc. (w/v %)**	8 % in HFIP	10 % in HFIP	8 % in HFIP	8 % in HFIP
**Dispensing Capillary ID**	125 µL 15 µL/min	125 µL 10 µL/min	350 µL 10 µL/min	350µL 10 µL/min
**Dispense Volume Dispense Rate**	0.62 mm (20G) Stainless Steel	0.62 mm (20G) Stainless Steel	0.62 mm (20G) Stainless Steel	0.81 mm (18G) Stainless Steel
**Nozzle Translation Distance & Rate**	80 mm 30 mm/s	80 mm 30 mm/s	80 mm 30 mm/s	80 mm 30 mm/s
**Mandrel OD**	1.1 mm	1.1 mm	1.1 mm	1.1 mm
**Mandrel Length**	150 mm	150 mm	150 mm	150 mm
**Mandrel Coating**	PTFE	PTFE	PTFE	PTFE
**Mandrel Rotation**	300 RPM	300 RPM	1000 RPM	1000 RPM
**Working Distance**	15 cm	12 cm	10 cm	12 cm
**Voltage Settings**	+15 kV Nozzle -1 kV Mandrel	+15 kV Nozzle -1 kV Mandrel	+20 kV Nozzle -4 kV Mandrel	+20 kV Nozzle -4 kV Mandrel
**Temp. & Humidity**	21°C, 30 %	21°C, 30 %	21°C, 30 %	21°C, 30 %
**Crosslinking**	None	0.5% Genipin 24h	None	None
**Portion Used**	Central 50 mm	Central 50 mm	Central 50 mm	Central 50 mm
